# Equilibrium Thermodynamics, Formation, and Dissociation Kinetics of Trivalent Iron and Gallium Complexes of Triazacyclononane-Triphosphinate (TRAP) Chelators: Unraveling the Foundations of Highly Selective Ga-68 Labeling

**DOI:** 10.3389/fchem.2018.00170

**Published:** 2018-05-23

**Authors:** Adrienn Vágner, Attila Forgács, Ernő Brücher, Imre Tóth, Alessandro Maiocchi, Alexander Wurzer, Hans-Jürgen Wester, Johannes Notni, Zsolt Baranyai

**Affiliations:** ^1^Department of Inorganic and Analytical Chemistry, University of Debrecen, Debrecen, Hungary; ^2^Bracco Imaging S.p.a., Bracco Research Centre, Colleretto Giacosa, Italy; ^3^Radiopharmaceutical Chemistry, Technische Universität München, Garching bei München, Germany

**Keywords:** chelates, gallium, iron, thermodynamics, kinetics, reaction mechanism, positron emission tomography

## Abstract

In order to rationalize the influence of Fe^III^ contamination on labeling with the ^68^Ga eluted from ^68^Ge/^68^Ga-*g*enerator, a detailed investigation was carried out on the equilibrium properties, formation and dissociation kinetics of Ga^III^- and Fe^III^-complexes of 1,4,7-triazacyclononane-1,4,7-tris(methylene[2-carboxyethylphosphinic acid]) (H_6_TRAP). The stability and protonation constants of the [Fe(TRAP)]^3−^ complex were determined by pH-potentiometry and spectrophotometry by following the competition reaction between the TRAP ligand and benzhydroxamic acid (0.15 M NaNO_3_, 25°C). The formation rates of [Fe(TRAP)] and [Ga(TRAP)] complexes were determined by spectrophotometry and ^31^P-NMR spectroscopy in the pH range 4.5–6.5 in the presence of 5–40 fold H_x_TRAP^(x−6)^ excess (x = 1 and 2, 0.15 M NaNO_3_, 25°C). The kinetic inertness of [Fe(TRAP)]^3−^ and [Ga(TRAP)]^3−^ was examined by the trans-chelation reactions with 10 to 20-fold excess of H_x_HBED^(x−4)^ ligand by spectrophotometry at 25°C in 0.15 M NaCl (x = 0,1 and 2). The stability constant of [Fe(TRAP)]^3−^ (log*K*_FeL_ = 26.7) is very similar to that of [Ga(TRAP)]^3−^ (log*K*_GaL_ = 26.2). The rates of ligand exchange reaction of [Fe(TRAP)]^3−^ and [Ga(TRAP)]^3−^ with H_x_HBED^(x−4)^ are similar. The reactions take place quite slowly via spontaneous dissociation of [M(TRAP)]^3−^, [M(TRAP)OH]^4−^ and [M(TRAP)(OH)_2_]^5−^ species. Dissociation half-lives (*t*_1/2_) of [Fe(TRAP)]^3−^ and [Ga(TRAP)]^3−^ complexes are 1.1 × 10^5^ and 1.4 × 10^5^ h at pH = 7.4 and 25°C. The formation reactions of [Fe(TRAP)]^3−^ and [Ga(TRAP)]^3−^ are also slow due to the formation of the unusually stable monoprotonated [^*^M(HTRAP)]^2−^ intermediates [^*^log*K*_Ga(HL)_ = 10.4 and ^*^log*K*_Fe(HL)_ = 9.9], which are much more stable than the [^*^Ga(HNOTA)]^+^ intermediate [^*^log*K*_Ga(HL)_ = 4.2]. Deprotonation and transformation of the monoprotonated [^*^M(HTRAP)]^2−^ intermediates into the final complex occur via OH^−^-assisted reactions. Rate constants (*k*_OH_) characterizing the OH^−^-driven deprotonation and transformation of [^*^ Ga(HTRAP)]^2−^ and [^*^Fe(HTRAP)]^2−^ intermediates are 1.4 × 10^5^ M^−1^s^−1^ and 3.4 × 10^4^ M^−1^s^−1^, respectively. In conclusion, the equilibrium and kinetic properties of [Fe(TRAP)] and [Ga(TRAP)] complexes are remarkably similar due to the close physico-chemical properties of Fe^III^ and Ga^III^-ions. However, a slightly faster formation of [Ga(TRAP)] over [Fe(TRAP)] provides a rationale for a previously observed, selective complexation of ^68^Ga^III^ in presence of excess Fe^III^.

## Introduction

Due to the wealth of obtainable information resulting in a high diagnostic value, medical imaging plays an ever-increasing role in modern personalized healthcare. In this context, radionuclide based imaging modalities which exploit George Hevesy's tracer principle (Levi, 1976) allow for unique functional diagnostics, because they enable monitoring of biological processes without significant interference with the investigated subject owing to minuscule amounts of administered active compound. Although the majority of nuclear imaging procedures (estimated >85%) still are scintigraphic or single photon emission computed tomography (SPECT) scans relying on the gamma-emitter ^99m^Tc, recent times have seen a strong surge in positron emission tomograpy (PET), following introduction of scanners capable of simultaneous functional and morphological imaging utilizing PET and computed tomography (CT) in 2001 (Beyer et al., 2000). While most PET investigations rely on the positron emitter ^18^F (more precisely, on the radiofluorinated glucose derivative [^18^F]2-fluoro-2-deoxy-d-glucose), some positron-emitting metal ion radionuclides have also received considerable attention in recent times (Wadas et al., 2010). Among these, ^68^Ga has arguably the highest value for preclinical and translational studies (Notni and Wester, 2018), mainly because it is obtained for a low price per dose from radionuclide generators. These small benchtop devices, which act as cyclotron-independent continuous on-site nuclide sources, contain ^68^Ge adsorbed on an inorganic matrix, such as SnO_2_ or TiO_2_, while decay of ^68^Ge produces ^68^Ga^III^ which can be eluted with dilute HCl (Notni, 2012; Rösch, 2013). Notably, such eluate frequently contains small amounts of impurities originating from the sorbent (Simecek et al., 2013), such as Ti^IV^ but also Fe^III^, Cu^II^, Zn^II^, or Al^III^ in form of their aqua or chlorido complexes.

**Scheme 1:**
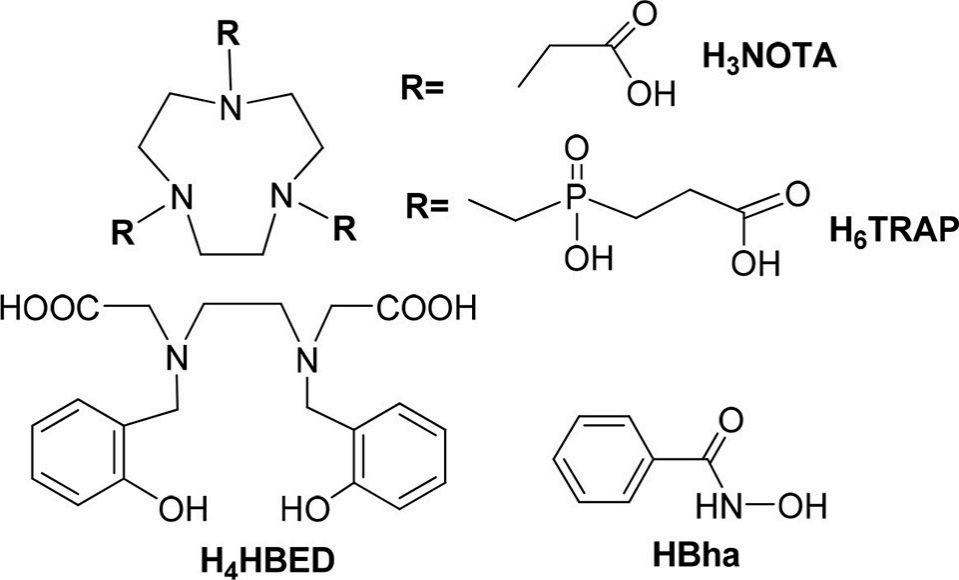
Structural formula of H_3_NOTA, H_6_TRAP, H_4_HBED and HBha chelates (H_3_NOTA: 1,4,7-triazacyclononane-1,4,7-triacetic acid; H_6_TRAP: 1,4,7-triazacyclononane-1,4,7-tris(methylene[2-carboxyethylphosphinic acid]); H_4_HBED: *N*, *N*′-Bis(2-hydroxybenzyl)ethylenediamine-*N*, *N*′-diacetic acid; HBha: benzhydroxamic acid).

^68^Ga-labeling of biomolecules usually requires prior decoration with a suitable multidentate ligand capable of binding the ^68^Ga^III^ ion into a kinetically inert complex (Wadas et al., 2010) and a plethora of ligands have been proposed for this purpose (Frank and Patrick, 2010; Velikyan, 2011). Against the background of aforementioned metal ion impurities in the generator eluate, an investigation of the radionuclide complexation efficiency of certain macrocycle-based chelators, among them TRAP (Notni et al., 2014) and NOTA (Mariko and Susumu, 1977; Scheme 1) pointed at a markedly different influence of non-Ga^III^ metal ions present in the ^68^Ga^III^ solutions used for radiolabeling (Simecek et al., 2013). In particular, TRAP was shown to tolerate much higher concentrations of Zn^II^, Cu^II^, and Fe^III^. Although highly similar structural features of [Fe(H_3_TRAP)] and [Ga(H_3_TRAP)] point at a close relation of both systems (Notni et al., 2010), it was found that even a threefold stoichiometric excess of Fe^III^ over TRAP or its mono-conjugable congener NOPO (Simecek et al., 2014) did not result in a significant decrease of ^68^Ga incorporation, whereas labeling of NOTA was almost completely inhibited. Particularly in view of the known similarity of Fe^III^ and Ga^III^, this discrepancy sheds a light on the mechanisms governing the superior ^68^Ga labeling properties of 1,4,7-triazacyclononanes bearing (methylene)phosphinic acid *N*-substituents (Notni et al., 2011). In order to gain a more detailed understanding, thermodynamics as well as formation and dissociation kinetic studies were performed for Ga^III^- and Fe^III^-complexes formed with TRAP and NOTA.

## Materials and methods

### Materials

The chemicals used for the experiments were of the highest analytical grade. Ga(NO_3_)_3_ and Fe(NO_3_)_3_ were prepared by dissolving Ga_2_O_3_ (99.9%, Fluka) and Fe_2_O_3_ (99.9% Fluka) in 6M HNO_3_ and evaporating of the excess acid. The solid Ga(NO_3_)_3_ and Fe(NO_3_)_3_ were dissolved in 0.1 M HNO_3_ solution. The concentration of the Ga(NO_3_)_3_ and Fe(NO_3_)_3_ solutions were determined by complexometry with the use of standardized Na_2_H_2_EDTA in excess. The excess of the Na_2_H_2_EDTA was measured with standardized ZnCl_2_ solution and xylenol orange as indicator. The H^+^ concentration of the Ga(NO_3_)_3_ and Fe(NO_3_)_3_ solutions was determined by pH potentiometric titration in the presence of Na_2_H_2_EDTA excess. The concentration of the H_6_TRAP, H_4_HBED, benzohydroxamic acid (HBha) and H_3_NOTA (provided by Prof. Petr Hermann, Department of Inorganic Chemistry, Faculty of Science, Charles University, Prague, Czech Republic) was determined by pH-potentiometric titration in the presence and absence of a large (40-fold) excess of CaCl_2_. All the measurements were made at constant ionic strength maintained by 0.15 M NaNO_3_ or NaCl at 25°C.

### Equilibrium studies

For determining the protonation constants of H_6_TRAP and H_3_NOTA ligands three parallel pH-potentiometric titration were made with 0.2 M NaOH in 0.002 M ligand solutions.

Stability constant of [Fe(Bha)]^2+^ complex was determined by spectrophotometry, studying the Fe^III^-HBha systems at the absorption band of Fe^III^-complex over the wavelength range of 400–800 nm in two sets of experiments. Individual samples were prepared in the first series in which the concentrations of Fe^III^ and HBha was constant 0.2 and 2.0 mM, while that of the H^+^ was varied between 0.04 and 1.0 mM (eight samples, Figure S1). The H^+^ concentration in the samples was adjusted by addition of calculated amounts of 2.0 M HNO_3_. The ionic strength was constant in the samples with [H^+^] < 0.15 M ([H^+^]+[Na^+^] = 0.15 M). Samples were kept at 25°C for a week. Absorbance values were determined at 11 wavelengths (400, 415, 430, 445, 460, 475, 490, 505, 520, 535, and 550 nm). In the second set, spectrophotometric titrations were done with samples containing HBha ligand in 2.0 mM concentration, whereas the concentration of Fe^III^ was varied between 0.1–0.3 mM (Figures S2–S4). The pH of the samples was adjusted using concentrated NaOH and HNO_3_ solutions in the pH range 1.7–11.0 (0.15 M NaNO_3_ and 25°C). For calculation of the equilibrium constants, the best fit of the absorbance–pH data was obtained by assuming formation of [Fe(Bha)]^2+^, [Fe(Bha)_2_]^+^, [Fe(Bha)_3_], and [Fe(Bha)_2_(OH)_2_]^−^ species (Figure S5). The molar absorptivity of [Fe(Bha)]^2+^, [Fe(Bha)_2_]^+^, [Fe(Bha)_3_] and [Fe(Bha)_2_(OH)_2_]^−^ species were also determined at the same 11 wavelengths in these experiments (Figure S6).

The stability constant of the [Fe(TRAP)]^3−^ complex has been determined by spectrophotometry, using competition reactions between HTRAP^5−^ and Bha^−^ for Fe^III^ at pH = 10.0. Concentration of [Fe(TRAP)]^3−^ was 0.2 mM, while that of HBha was varied between 0.0 and 1.5 mM (6 samples). The samples were kept at 25°C for 2 weeks. Absorbance values of the Fe^III^-HTRAP^5−^-Bha^−^ systems were determined at 11 wavelengths (400, 415, 430, 445, 460, 475, 490, 505, 520, 535, and 550 nm). The molar absorptivities of [Fe(TRAP)]^3−^ and [Fe(TRAP)OH]^4−^ in equilibrium solutions were determined by recording the absorption spectra of 0.1, 0.2, and 0.3 mM solution of [Fe(TRAP)]^3−^ in the pH range 6.0–12.0. The molar absorptivity of [Fe(Bha)_2_(OH)_2_]^−^ species was determined in the separate experiments. Absorbance and pH values were determined in the samples after equilibration (the time needed to reach the equilibria was determined by spectrophotometry). Spectrophotometric measurements were done using 1.0 cm cells with a Cary 1E spectrophotometer at 25°C. Protonation constants of the Fe^III^ complex formed with TRAP^6−^ were determined by direct pH-potentiometric titration at 1:1 metal to ligand ratios (both concentrations were 0.002 M). For calculation of the log*K*_MHiL_ values, the mL base–pH data used were measured in the pH range 1.7 −12.0.

For pH measurements and titrations, a *Metrohm 785 DMP Titrino* titration workstation and a *Metrohm-6.0233.100* combined electrode were used. Equilibrium measurements were carried out at a constant ionic strength (0.15 M NaNO_3_ or NaCl) in 6 mL samples at 25°C. Solutions were stirred and continuously purged with N_2_. Titrations were performed in a pH range of 1.7–12.0. KH-phthalate (pH = 4.005) and borax (pH = 9.177) buffers were used to calibrate the pH meter. For calculation of [H^+^] from measured pH values, the method proposed by Irving et al. was used (Irving et al., 1967). A 0.01 M HNO_3_ or HCl solution was titrated with the standardized NaOH solution in the presence of 0.15 M NaNO_3_ or NaCl. Differences between the measured (pH_read_) and calculated pH (–log[H^+^]) values were used to obtain the equilibrium H^+^ concentration from the pH values, measured in the titration experiments. For equilibrium calculations, the stoichiometric water ionic product (p*K*_w_) is also needed to calculate [H^+^] values in basic conditions. The V_NaOH_–pH_read_ data pairs of the HNO_3_–NaOH or HCl–NaOH titration obtained in the pH range 10.5–12.0 have been used to calculate the p*K*_w_ value (p*K*_w_ = 13.84). For calculation of the equilibrium constants, the program PSEQUAD (Zekany and Nagypal, 1985) was used. The standard deviation (SD) of the equilibrium parameters calculated by the program PSEQUAD is defined by Equation (1)

(1) $$\text{SD}=\sqrt{\frac{\sum_{\text{j}=1}^{\text{j}=N}{\text{res}}_{\text{j}}^{\text{2}}}{\text{N}-\text{m}}}\times \sqrt{{\left[{\left({\text{J}}^{\text{T}}\cdot \text{J}\right)}^{-\text{1}}\right]}_{\text{ii}}}$$

where res, N, m, J and J^T^ are the residual, number of fitted data, number of refined parameters, Jacobian matrix and the transpose of Jacobian matrix, respectively.

### Kinetic studies

#### Formation kinetics of [Fe(TRAP)] and [Ga(TRAP)]

Formation rates of [Fe(TRAP)] were studied by spectrophotometry at 260 nm in the pH range of about 4.5–6.5. Kinetic studies were carried out with *Cary 1E* and *Cary 100 Bio* spectrophotometers, using cell holders thermostated to 25°C. The pre-thermostated solutions were mixed in tandem cells (l = 0.874 cm). Formation of Fe^III^ complexes were studied in the presence of a 5- to 40-fold ligand excess in order to maintain pseudo-first-order conditions ([Fe^III^] = 0.1 mM). Pseudo-first-order rate constants (*k* = *k*_obs_) were calculated by fitting the absorbance values to the equation:

(2) $${\text{A}}_{\text{t}}=({\text{A}}_{\text{0}}-{\text{A}}_{\text{e}}){\text{e}}^{\left(-\text{kt}\right)}{+\text{A}}_{\text{e}}$$

wherein *A*_0_, *A*_*e*_, and *A*_*t*_ are the absorbance values at the start (*t* = 0 s), at equilibrium and at the time *t* of the reaction, respectively. Formation of [Ga(TRAP)]^3−^ was monitored by ^31^P-NMR spectroscopy on the signal of the forming Ga(TRAP) complex. ^31^P-NMR spectra were recorded by a Bruker DRX 400 spectrometer (^31^P, 161.97 MHz, 9.4 T) equipped with Bruker VT-1000 thermocontroller, using a 5 mm broad band probe. Kinetic experiments were performed at a constant temperature of 25.0°C. The formation rates were studied in the pH range of about 4.5–6.3. For these experiments, Ga(NO_3_)_3_ and H_6_TRAP solutions were prepared in H_2_O (a capillary with D_2_O was used for lock). In all experiments, the concentration of Ga^III^ was 1 mM, while that of the H_6_TRAP was varied between 5 and 30 fold excess in order to maintain pseudo-first-order conditions. Pseudo-first-order rate constants (*k* = *k*_obs_) were calculated by fitting the integral signal values to the Equation (2). The ionic strength of the solutions was kept constant at 0.15 M with NaNO_3_. To keep the pH values constant, *N*-methylpiperazine (pH range of 4.1–5.2) and piperazine (pH range of 4.7–6.6) buffers (0.01 M) were used.

#### Dissociation kinetics of Fe(TRAP) and Ga(TRAP)

The rates of the ligand exchange reactions of Fe(TRAP) and Ga(TRAP) with H_x_HBED^x−4^ (x = 0,1 and 2) ligand were studied by following the formation of [Fe(HBED)]^−^ and [Ga(HBED)]^−^ complexes by spectrophotometry at 470 nm and 290 nm, respectively. All experiments were performed in the presence of 10- and 20-fold excess of H_x_HBED^x−4^ (x = 1 and 2) in order to maintain pseudo-first order kinetic conditions ([Fe(TRAP)] = [Ga(TRAP)] = 0.2 mM). The pseudo-first-order rate constants (k = k_d_) were calculated by fitting the absorbance values to the Equation (2). Kinetic studies were performed with Cary 1E and Cary 100 Bio spectrophotometers, using cell holders thermostated to 25°C. The pre-thermostated solutions were mixed in tandem cells (l = 0.874 cm). The ionic strength of the solutions was kept constant at 0.15 M with NaCl. The ligand exchange reactions were followed at 25°C in the pH range 9.0–14.0. The OH^−^ concentration at pH > 12 was adjusted by addition of calculated amounts of 4.0 M NaOH solution. Buffers were not used to keep the pH constant due to the high buffer capacity of the H_x_HBED^x−4^ (x = 1 and 2) excess at pH < 12. Calculation of the kinetic parameters was performed with the Micromath Scientist computer program (version 2.0, Salt Lake City, UT, USA).

## Results and discussion

### Solution thermodynamics

Protonation equilibria of the TRAP^6−^, NOTA^3−^ and Bha^−^ ligands were studied by pH-potentiometry. The protonation constants (log$K_{\text{i}}^{\text{H}}$) of ligands defined by Equation (3) are listed in Table 1 (standard deviations are shown in parentheses). The charges of ligands and complexes will be indicated when it is necessary.

(3) $${\text{K}}_{\text{i}}^{\text{H}}=\frac{\left[{\text{H}}_{\text{i}}\text{L}\right]}{\left[{\text{H}}_{\text{i}-\text{1}}\text{L}][{\text{H}}^{+}\right]}\text{ i}=\text{0},\text{1},\text{2}\ldots \text{6}$$

The protonation schemes of TRAP^6−^ and NOTA^3−^ ligands were well characterized by both spectroscopic and potentiometric methods (Bevilacqua et al., 1987; Geraldes et al., 1991; Notni et al., 2010). These studies reveal that the first and second protonations occur at two ring nitrogen atoms, whereas the third, fourth and fifth protonations occur at the carboxylate groups of NOTA^3−^ and TRAP^6−^. The sixth proton of the TRAP^6−^ ligand binds on the phosphinate oxygen atom. Interestingly, not all phosphinate groups are protonated, even under very acidic conditions (pH < 1), which is why they are still able to coordinate to metal ions. A comparison of protonation constants of TRAP^6−^ and NOTA^3−^ indicates that log$K_{1}^{\text{H}}$ value of TRAP^6−^ is significantly lower than that of NOTA^3−^ (Table 1). The lower first protonation constant of TRAP^6−^ can be attributed to formation of a weaker H-bond between the protonated ring nitrogen and the phosphinate oxygens than that formed between the protonated ring nitrogen and the carboxylate oxygens in HNOTA^2−^. Comparison of the protonation constants obtained in 0.15 M NaNO_3_ or NaCl, 0.1 M KCl and 0.1 M Me_4_NCl solutions indicates that the log$K_{\text{i}}^{\text{H}}$ values of TRAP^6−^ are independent of the ionic strength, whereas the log$K_{1}^{\text{H}}$ value of NOTA^3−^ is significantly lower in the presence of K^+^ and Na^+^ ions, which can be attributed to formation of [K(NOTA)]^2−^ and [Na(NOTA)]^2−^ complexes. Total basicity of ligands (Σlog$K_{\text{i}}^{\text{H}}$, Table 1) generally correlates with the stability constants (*K*_ML_) of their metal complexes. (For the calculation of Σlog$K_{\text{i}}^{\text{H}}$ value of TRAP^6−^, the log$K_{\text{i}}^{\text{H}}$ values of the carboxylate groups were not considered because they do not participate in the coordination of metal ions). The Σlog$K_{\text{i}}^{\text{H}}$ values (Table 1) show that the total basicity of TRAP^6−^ is significantly lower than that of NOTA^3−^ because of the lower protonation constant of the ring nitrogen (log$K_{1}^{\text{H}}$) and phosphinate oxygen atoms of the TRAP^6−^ ligand. Therefore, lower stability constants should be expected for the TRAP^6−^ complexes than those of NOTA^3−^ complexes.

**Table 1:** Protonation constants of TRAP^6−^, NOTA^3−^, and Bha^−^ ligands (25°C).

	**I**	**log$K_{1}^{\text{H}}$**	**log$K_{2}^{\text{H}}$**	**log$K_{3}^{\text{H}}$**	**log$K_{4}^{\text{H}}$**	**log$K_{5}^{\text{H}}$**	**log$K_{6}^{\text{H}}$**	**Σlog$K_{\text{i}}^{\text{Hf}}$**
TRAP^6−^	0.15 M NaNO_3_	11.60(2)	5.39(2)	4.42(2)	4.19(3)	3.46(3)	1.60(2)	18.59 ^g^
	0.15 M NaCl^a^	11.74	5.46	4.80	4.16	3.49	1.50	18.70 ^g^
	0.1 M Me_4_NCl ^b^	11.48	5.44	4.84	4.23	3.45	1.66	18.58 ^g^
NOTA^3−^	0.15 M NaNO_3_	11.94(2)	5.71(3)	3.14(3)	1.60(2)	–	–	22.39
	0.15 M NaCl^a^	12.16	5.75	3.18	1.90	–	–	22.99
	0.1 M KCl^c^	11.98	5.65	3.18	–	–	–	–
	0.1 M Me_4_NCl^d^	13.17	5.74	3.22	1.96	–	–	24.09
Bha^−^	0.15 M NaNO_3_	8.53(3)	–	–	–	–	–	–
	0.2 M KCl^e^	8.69	–	–	–	–	–	–

a Ref. (Baranyai et al., 2015);

b Ref. (Notni et al., 2010);

c Ref. (Clarke and Martell, 1991);

d Ref. (Drahos et al., 2011);

e Ref. (Farkas et al., 1998);

f Total ligand basicity (Σlog$k_{i}^{H}$) characterizes the sum of basicity of donor atoms;

g *The protonation constants of the acetate pendants (log$k_{3}^{H}$, log$k_{4}^{H}$ and log$k_{5}^{H}$) of TRAP^6−^ were not considered in the calculation of Σlog$k_{i}^{H}$ values*.

Stability and protonation constants of TRAP^6−^ and NOTA^3−^ complexes formed with Fe^III^ were determined by pH-potentiometry and UV/Vis spectrophotometry. The stability and protonation constants of the metal complexes formed with the TRAP^6−^ and NOTA^3−^ ligands listed in Table 2 are defined by Equations (4–6):

(4) $$\begin{array}{l} {\text{M}}^{\text{III}}+\text{L}⇌\text{ML}\\ {\text{K}}_{\text{ML}}=\frac{\left[\text{ML}\right]}{\left[\text{M}][\text{L}\right]} \end{array}$$

(5) $$\begin{array}{l} {\text{MH}}_{\text{i}-\text{1}}\text{L}+{\text{H}}^{+}⇌{\text{MH}}_{\text{i}}\text{L}\\ {\text{K}}_{{\text{MH}}_{\text{i}}\text{L}}=\frac{\left[{\text{MH}}_{\text{i}}\text{L}\right]}{\left[{\text{MH}}_{\text{i}-\text{1}}\text{L}][{\text{H}}^{+}\right]} \end{array}$$

(6) $$\begin{array}{l} \text{M}(\text{L})\text{OH}+{\text{H}}^{+}⇌\text{ML}\\ {\text{K}}_{\text{M}(\text{L})\text{OH}}=\frac{\left[\text{ML}\right]}{\left[\text{M}(\text{L})\text{OH}][{\text{H}}^{+}\right]} \end{array}$$

wherein i = 1, 2, or 3. Since the [Fe(TRAP)]^3−^ and [Fe(NOTA)] complexes are highly stable, formation of Fe^III^ complexes was practically completed at about pH < 2.0. Therefore, from the data obtained by pH-potentiometric titrations performed at 1:1 metal to ligand concentration ratio, only the protonation constants of the [Fe(TRAP)]^3−^ and [Fe(NOTA)] complexes could be calculated. In order to determine the log*K*_FeL_ value, we studied the competition reactions between HTRAP^5−^ and Bha^−^ for Fe^III^ [Equation (7)] by spectrophotometry in the wavelength range 400–800 nm. To calculate the stability constant of [Fe(TRAP)]^3−^, the equilibrium constants characterizing the species formed in the Fe^III^-HBha system have been determined from the data obtained by pH-potentiometric and spectrophotometric measurements (experimental detail and calculation procedures used for the characterization of Fe^III^-HBha system are summarized in the Supplementary information).

**Table 2:** Stability and protonation constants (log*K*) of Fe^III^ and Ga^III^-complexes formed with TRAP^6−^, NOTA^3−^, and Bha^−^ ligand (25°C).

	**TRAP**^**6−**^		**NOTA**^**3−**^		**Bha**^**−**^	
	**Fe^III^**	**Ga^III^**	**Fe^III^**	**Ga^III^**	**Fe**^**III**^	
I	0.15 M NaNO_3_	0.1 M Me_4_NCl^a^	0.1 M KCl^b^	0.1 M Me_4_NCl^c^	0.15 M NaNO_3_	0.2 M KCl^d^
ML	26.73(8)	26.24	28.3	29.60	10.80(2)	11.08
MHL	5.07(2)	5.18	–	0.9	–	–
MH_2_L	4.34(2)	4.55	–	–	–	–
MH_3_L	3.20(2)	3.77	–	–	–	–
MH_4_L	–	0.7	–	–	–	–
M(L)OH	9.76(2)	9.84	9.12(4)^e^	9.83	–	–
ML_2_	–	–	–	–	9.03(2)	10.12
ML_3_	–	–	–	–	7.41(3)	7.60
logβ_FeL2(OH)2_	–	–	–	–	6.68(5)	–

a Ref. (Notni et al., 2010);

b Ref. (Clarke and Martell, 1991);

c Ref. (Simecek et al., 2012);

d Ref. (Farkas et al., 1998);

e *In this work (0.15 M NaNO_3_, 25°C)*.

(7) $$\begin{array}{l} {\left[\text{Fe}(\text{TRAP}){\left(\text{OH}\right)}_{\text{x}}\right]}^{\left(-\text{3}-\text{x}\right)}{\text{+Bha}}^{-}⇌{\left[\text{Fe}{\left(\text{Bha}\right)}_{\text{2}}{\left(\text{OH}\right)}_{\text{2}}\right]}^{-}\\ {\text{+HTRAP}}^{\text{5}-} \end{array}$$

wherein x = 0 and 1. The pH of the samples was 10.0, when [Fe(TRAP)]^3−^, [Fe(TRAP)OH]^4−^ and [Fe(Bha)_2_(OH)_2_]^−^ were formed. Some characteristic absorption spectra of Fe^III^-HTRAP^5−^Bha^−^ systems are shown in Figure 1.

**Figure 1:**
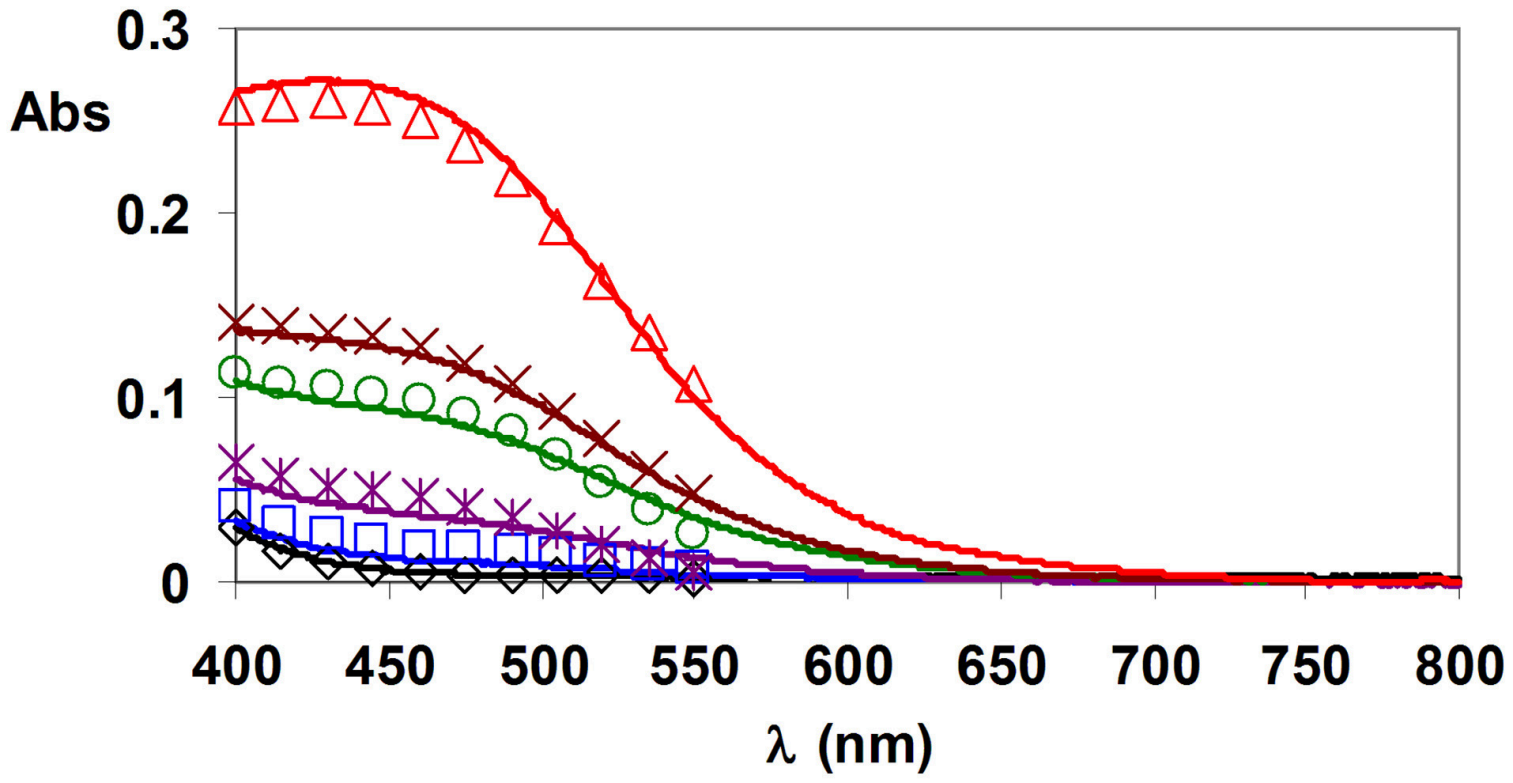
Absorption spectra of Fe^III^–HTRAP^5−^-Bha^−^ equilibrium systems. Open symbols and solid lines represent experimental and calculated absorbance values, respectively. ([Fe(TRAP)(OH)_*x*_^(−3−x)^] = 0.2 mM, [HBha] = **0.0** mM (black). 0.25 mM (blue), 0.5 mM (purple), 0.75 mM (green), 1.0 mM (brown), and 1.5 mM (red), x = 0 and 1, pH = 10.0, 0.15 M NaNO_3_, 25°C).

The stability and protonation constants of [Fe(TRAP)]^3−^ complex have been calculated by the combination of the pH-potentiometric data obtained by the titration of [Fe(TRAP)]^3−^ complex with NaOH solution in the pH range 1.7–12.0 (Figure S7) with the spectrophotometric data acquired at pH = 10.0 in Fe^III^-HTRAP^5−^–Bha^−^ system (Figure 1). For calculation of the log*K*_FeL_ value, protonation constants of Bha^−^ (Table 1), the stability constant (Table 2) and the molar absorptivity of the [Fe(Bha)_2_(OH)_2_]^−^ complex were used. Stability and protonation constants obtained for [Fe(TRAP)]^3−^ are shown in Table 2.

Comparison of stability constants in Table 2 reveals that the log*K*_ML_ values of [Fe(TRAP)]^3−^ and [Ga(TRAP)]^3−^ complexes are essentially equal and 2–3 log*K* unit smaller than those of the corresponding NOTA^3−^ complexes. The higher stability constant of [Fe(NOTA)] and [Ga(NOTA)] complexes can be attributed to higher total basicity of NOTA^3−^. The stability constant of [Fe(NOTA)] is about one log*K* unit lower than that of [Ga(NOTA)], which corresponds to a lower log$K_{1}^{\text{H}}$ value of NOTA^3−^ obtained in 0.1 M KCl solution. The triazacyclononane macrocyclic ligands with carboxylate or phosphinate pendant arms show similar affinity to Fe^III^ and Ga^III^, which is readily explained by the facts that Ga^3+^ and Fe^3+^ have similar ionic radii (0.62 Å and 0.65 Å, respectively), and share the same charge and preferred coordination number (CN = 6).

**Figure 2:**
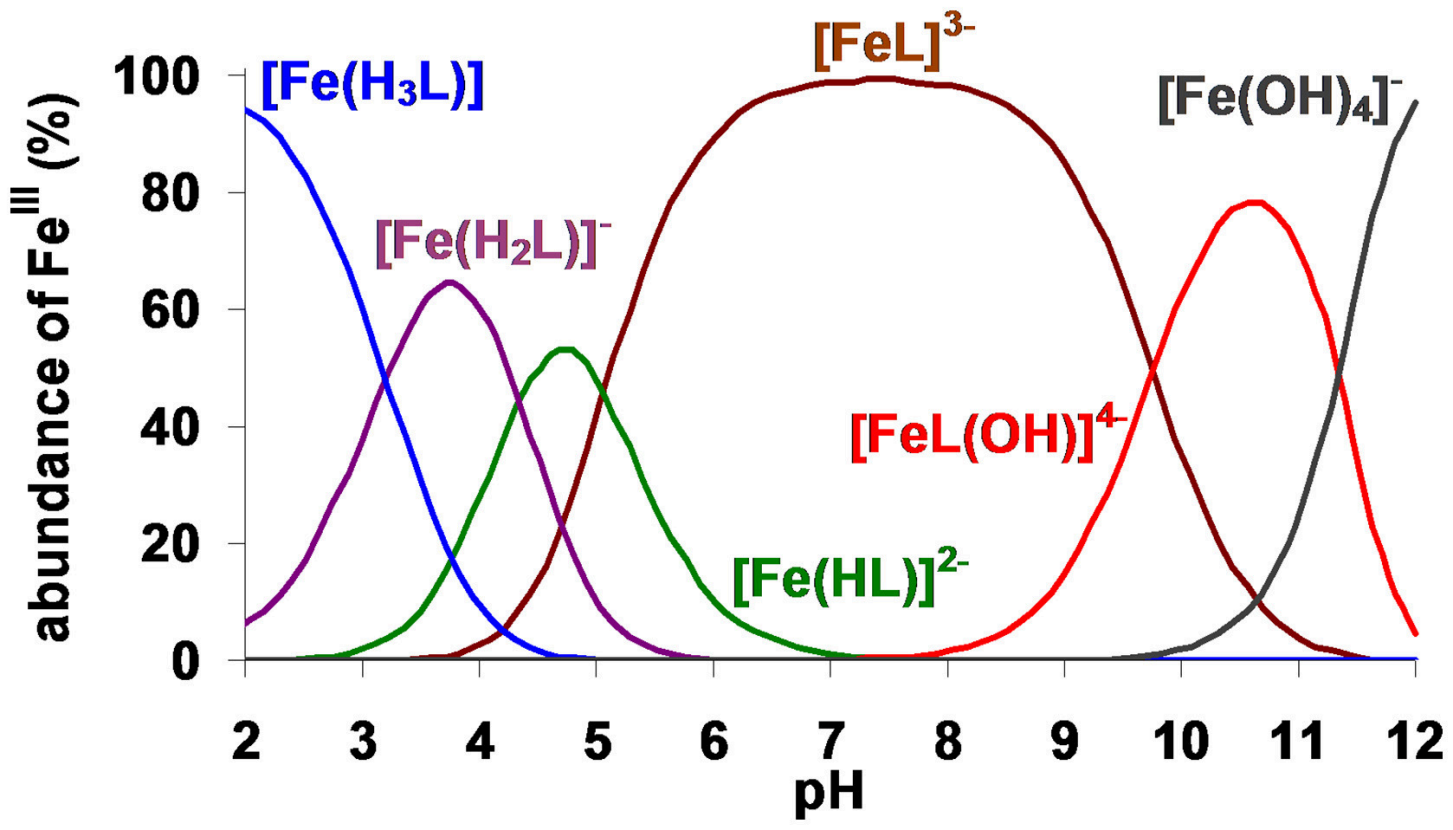
Species distribution of Fe^III^ – TRAP^6−^ system ([Fe^III^] = [TRAP^6−^] = 0.2 mM, 0.15 M NaNO_3_, 25°C).

The species distribution diagram of the Fe^III^-TRAP^6−^ system (Figure 2) shows that the Fe^III^ complex is fully formed even at pH < 2 in the form of a tri-protonated [Fe(H_3_L)] species. Upon rising the pH from 2.0 to 7.0, stepwise deprotonation results in consecutive formation of [Fe(H_2_L)]^−^ and [Fe(HL)]^2−^. Since the protonation constants characterizing the formation of the [Fe(HL)]^2−^, [Fe(H_2_L)]^−^ and [Fe(H_3_L)] species are very similar to the log$K_{3}^{\text{H}}$, log$K_{4}^{\text{H}}$ and log$K_{5}^{\text{H}}$ values of the free TRAP^6−^ ligand, [Fe(TRAP)]^3−^ is protonated on the non-coordinating carboxylate pendant arms. According to the known solid state structures of [Fe(H_3_TRAP)], the coordination environment of Fe^III^ is characterized by the trigonal antiprismatic structure formed by the parallel ring-N_3_ and phosphinate-O_3_ planes, whereas the carboxylate groups are protonated and non-coordinated (the solid state structure of [Ga(H_3_TRAP)] complex is very similar to that of [Fe(H_3_TRAP)]) (Notni et al., 2010). The [Fe(TRAP)]^3−^ complex predominates in the pH range 6.0–9.0. The pH-potentiometric titration data, obtained at pH > 8 for [Fe(TRAP)]^3−^, indicate a base-consuming process, which can be attributed to substitution of one of the phosphinate oxygens with a OH^−^ ion in the coordination sphere of Fe^III^ upon formation of the [Fe(TRAP)OH]^4−^ species [Equation (6)]. Similar processes were also identified for [Ga(TRAP)]^3−^, [Fe(NOTA)] (Figure S8 and Table 2) and [Ga(NOTA)] complexes (Notni et al., 2010; Simecek et al., 2012).

### Formation kinetics of Fe(TRAP) and Ga(TRAP) complexes

The formation reactions between NOTA and various metals, such as lanthanide(III) ions (Ln^III^) but also Ga^III^, are typically slow at pH around 2.0–5.0 (Brucher and Sherry, 1990; Morfin and Toth, 2011). Since formation of Ln^III^ and Ga^III^ complexes of open-chain ligands is generally fast, the slow formation kinetics of the NOTA complexes can be attributed to the rigidity of the triaza-cyclononane macrocycle. Incorporation of Ln^III^- and Ga^III^-ions into the preformed coordination cage of NOTA is slow because of formation of stable mono-protonated [^*^Ln(HNOTA)]^+^ and [^*^Ga(HNOTA)]^+^ intermediates, which has been confirmed earlier by spectrophotometry measurements (Brucher and Sherry, 1990) and ^1^H NMR spectroscopy(Morfin and Toth, 2011). Stability constants of such intermediates have furthermore been determined from kinetic data obtained by spectrophotometry (Brucher and Sherry, 1990) and ^1^H NMR spectroscopy (Morfin and Toth, 2011). In the intermediate, the proton is most likely attached to a macrocyclic nitrogen, and the electrostatic repulsion between the proton and a Ln^III^- or Ga^III^-ion can inhibit fast entrance of the metal ion into the coordination cage. Formation rates of the [Ln(NOTA)] and [Ga(NOTA)] complexes are directly proportional to the OH^−^ concentration, meaning that a rate-determining OH^−^ assisted deprotonation and rearrangement of the monoprotonated intermediate is followed by entrance of the Ln^III^- or Ga^III^-ion into the N_3_O_3_ coordination cage of NOTA^3−^(Brucher and Sherry, 1990; Morfin and Toth, 2011).

**Scheme 2:**
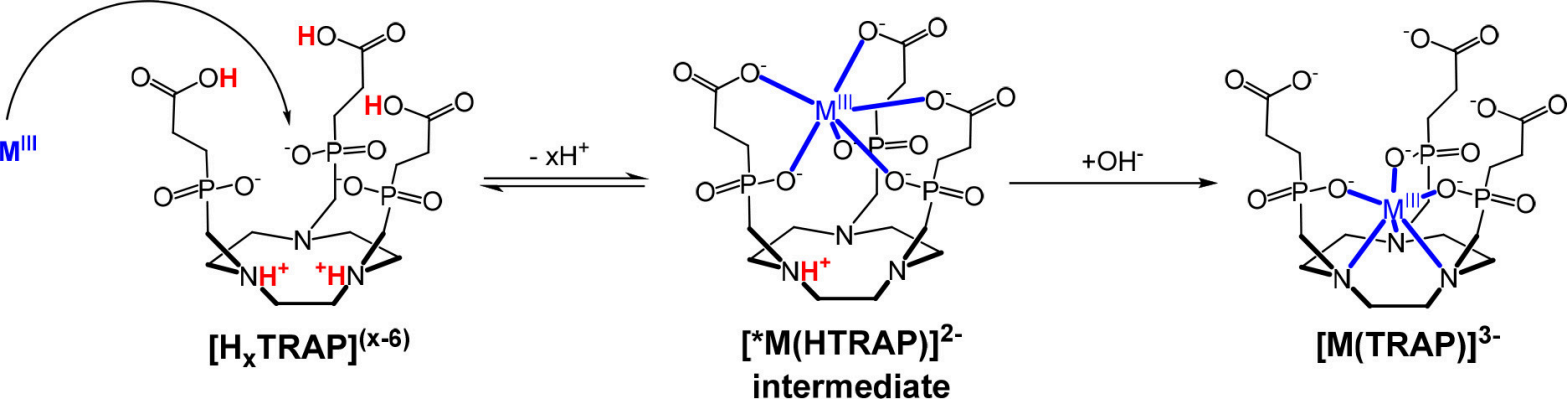
Formation mechanism of [M(TRAP)]^3−^ complexes.

In the present work, formation kinetics of M(TRAP) complexes (M^III^ = Fe^III^ and Ga^III^) have been studied by spectrophotometry on the absorption band of the forming Fe(TRAP) (λ = 260 nm) and by ^31^P-NMR spectroscopy following the integral value of the forming Ga(TRAP) complex in the pH range 4–6. UV-absorption as well as ^31^P-NMR spectra, recorded after mixing of solutions containing Fe(NO_3_)_3_ or Ga(NO_3_)_3_ with HTRAP^5−^ as functions of time, are shown in Figures S9, S10. For the reaction mixture of Fe^III^-HTRAP^5−^ at pH = 6.0, the absorption band observed between λ = 245–320 nm (Figure S9) can be explained by the formation of the intermediate. The absorbance values in the λ = 250–280 nm range increase with time, allowing for the conclusion that the intermediate is transformed into the final [Fe(TRAP)]^3−^ in-cage complex. Formation of the intermediate in Ga^III^-TRAP reactions mixtures was previously proven by ^31^P- and ^71^ Ga-NMR spectroscopy (Notni et al., 2010). Based on the similarity of TRAP and NOTA, it can be assumed that protonation of the ring nitrogen below pH = 10.0 initially hampers the formation of in-cage TRAP complexes while the three carboxylate and three phosphinate oxygen atoms of HTRAP^5−^ can be coordinated to the metal ions to form a mono-protonated [^*^M(HTRAP)]^2−^ intermediate, in which the Fe^III^ and Ga^III^ -ion is situated outside of the coordination cage. To complete the complex formation, the proton has to be removed from the ring nitrogen via a OH^−^-assisted reaction, followed by the rearrangement of the intermediate to the final [Fe(TRAP)]^3−^ and [Ga(TRAP)]^3−^ complexes (Scheme 2).

**Figure 3:**
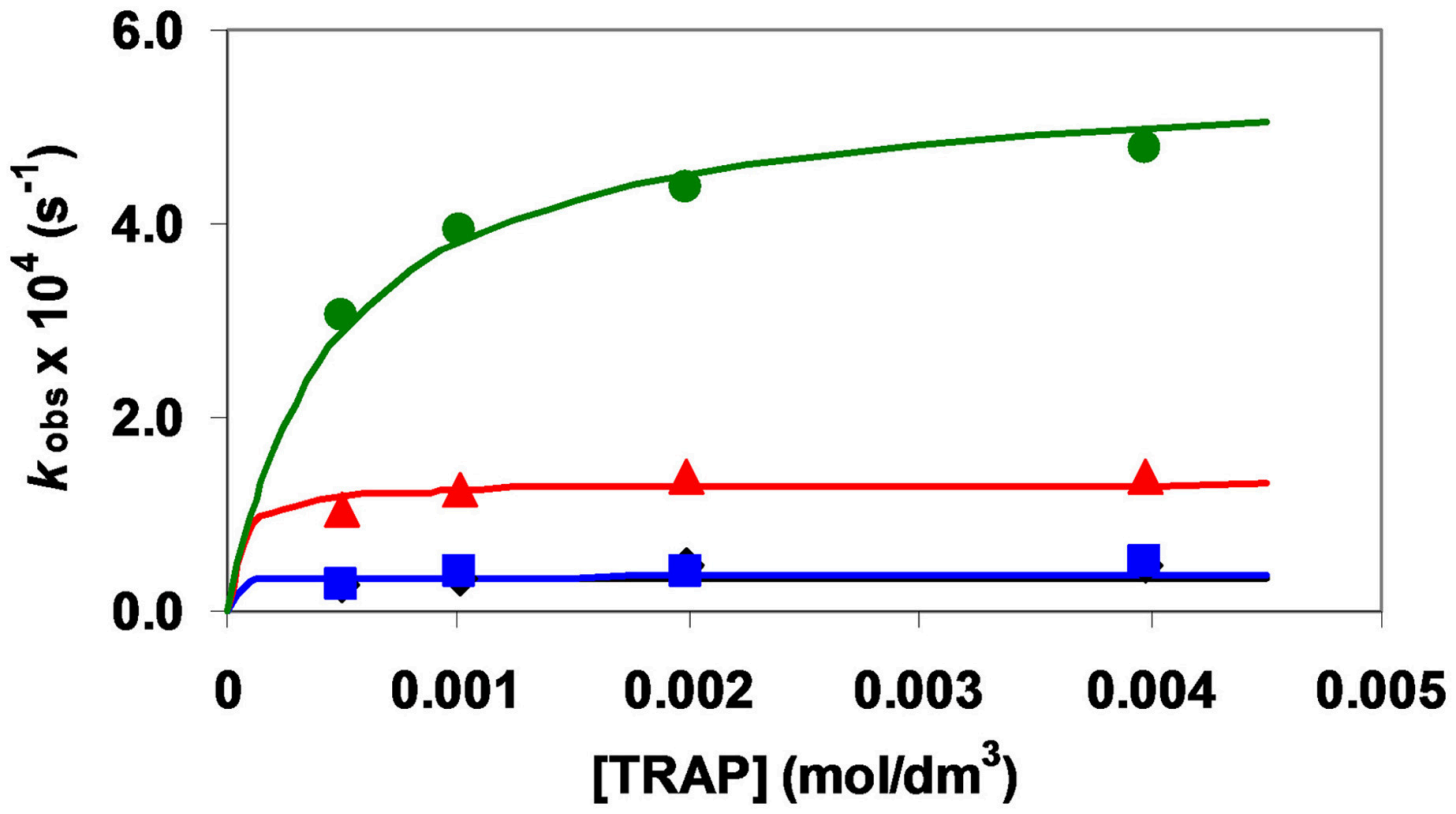
*k*_obs_ pseudo-first order rate constants for the formation reaction of [Fe(TRAP)]^3−^ as function of [H_x_TRAP]_t_ ([Fe^III^] = 0.1 mM, pH = 4.5 (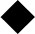), 5.0 (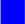), 5.5 (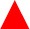), and 6.0 (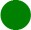), x = 1 and 2, 0.15 M NaNO_3_ and 25°C).

**Figure 4:**
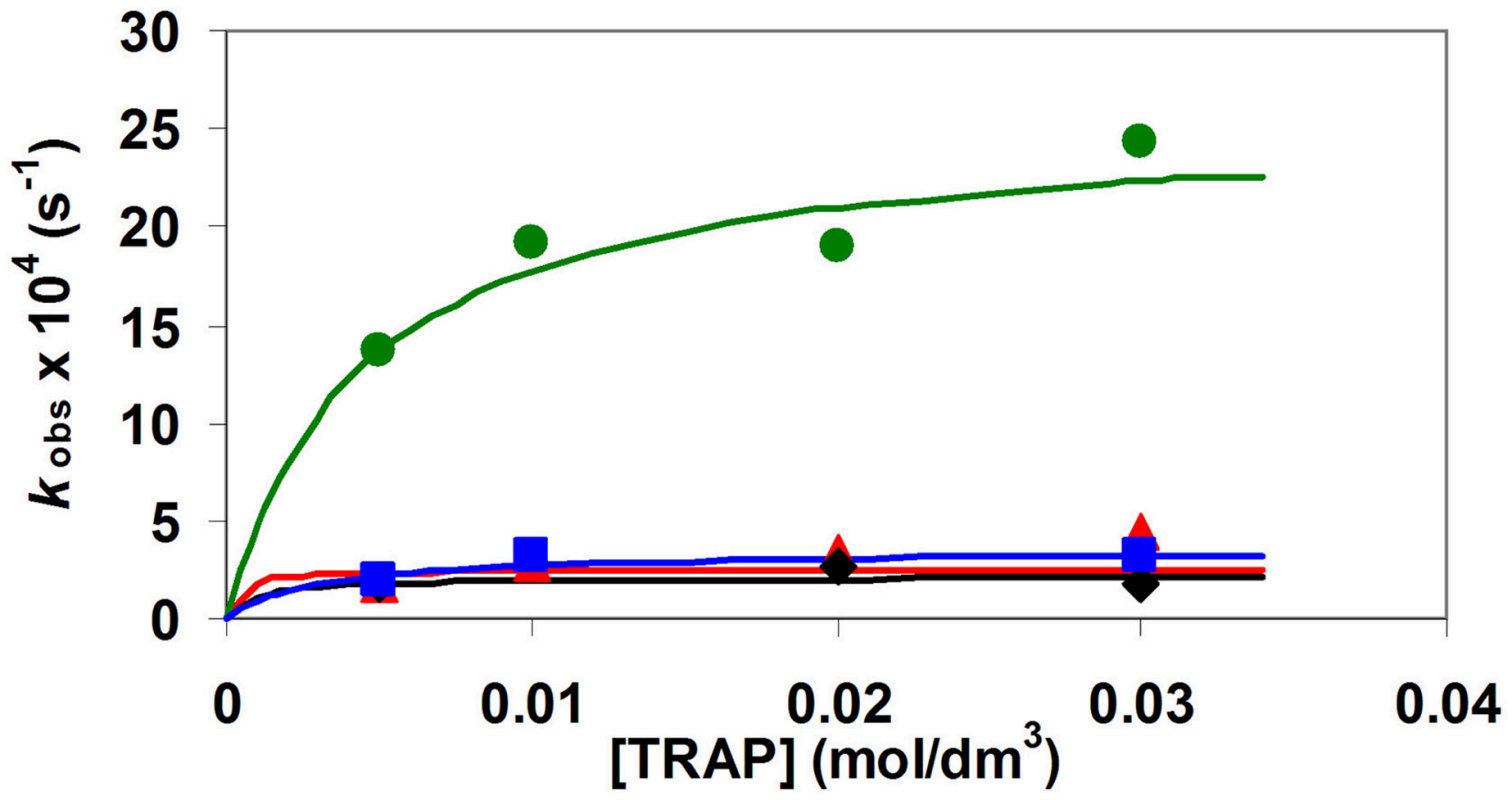
*k*_obs_ pseudo-first order rate constants for the formation reaction of [Ga(TRAP)]^3−^ as a function of [H_x_TRAP]_t_ ([Ga^III^] = 1 mM, pH = 4.6 (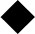), 5.0 (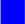), 5.6 (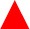), and 6.0 (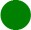), x = 1 and 2, 0.15 M NaNO_3_ and 25°C).

The formation rates of [Fe(TRAP)]^3−^ and [Ga(TRAP)]^3−^ have been studied under pseudo-first-order conditions in the presence of high excess of H_x_TRAP^(x−6)^ ([Fe^III^] = 1.0 × 10^−4^ M; [TRAP]_t_ = 0.5–4.0 × 10^−3^ M; [Ga^III^] = 1.0 × 10^−3^ M; [H_x_TRAP]_t_ = 5.0–30 × 10^−3^ M, x = 1 and 2). Under such conditions the rate of formation reactions can be expressed by Equation (8).

(8) $$\frac{\text{d}{\left[\text{ML}\right]}_{\text{t}}}{\text{dt}}={\text{k}}_{\text{obs}}{\left[{\text{M}}^{\text{III}}\right]}_{\text{t}}$$

wherein [ML]_t_ is the concentration of the [Fe(TRAP)]^3−^ and [Ga(TRAP)]^3−^ complexes, [M^III^]_t_ is the total concentration of species containing the Fe^III^ and Ga^III^ ions not bound to the H_x_TRAP^(x−6)^ ligand, and *k*_obs_ is a pseudo-first-order rate constant. As expected, the *k*_obs_ vs. [H_x_TRAP]_t_ curves (Figures 3, 4) are saturation curves indicating the formation of the [^*^M(HTRAP)]^2−^ intermediates characterized by the stability constant defined by Equation (9).

(9) $$*{\text{K}}_{\text{M}(\text{HL})}=\frac{{[}^{*}\text{M}(\text{HTRAP})]}{\left[{\text{M}}^{\text{III}}][\text{HTRAP}\right]}$$

The rate-determining step of the reactions is the deprotonation and rearrangement of the [^*^M(HTRAP)]^2−^ intermediates followed by the entrance of the metal ion into the coordination cage of the TRAP^6−^ ligand:

(10) $$\begin{array}{l} \frac{\text{d}{\left[\text{ML}\right]}_{\text{t}}}{\text{dt}}={\text{k}}_{\text{obs}}{\left[{\text{M}}^{\text{III}}\right]}_{t}={\text{k}}_{\text{f}}{[}^{*}\text{M}(\text{HTRAP})]\\ \;={\text{k}}_{\text{f}}{}^{*}{\text{K}}_{\text{M}(\text{HTRAP})}[{\text{M}}^{\text{III}}][\text{HTRAP}] \end{array}$$

wherein [^*^M(HTRAP)] is the concentration of [^*^M(HTRAP)]^2−^ intermediate and *k*_f_ is the rate constant characterizing the deprotonation and rearrangement of the intermediate to the [M(TRAP)]^3−^ complex. In the pH range studied, the concentration of the non-complexed ligand ([TRAP]_free_) can be expressed by Equation (11) using the protonation constants of TRAP^6−^ ligand (Table 1).

(11) $$\begin{array}{l} {\left[\text{TRAP}\right]}_{\text{free}}=\left[\text{HTRAP}\right](\text{1+}K_{\text{2}}^{\text{H}}\left[{\text{H}}^{+}\right]+K_{\text{2}}^{\text{H}}K_{\text{3}}^{\text{H}}{\left[{\text{H}}^{+}\right]}^{2}+\ldots \\ \text{ +}K_{\text{2}}^{\text{H}}K_{\text{3}}^{\text{H}}K_{\text{4}}^{\text{H}}K_{\text{5}}^{\text{H}}K_{\text{6}}^{\text{H}}{\left[{\text{H}}^{+}\right]}^{5})=(\text{1+}\alpha_{\text{H}})[\text{HTRAP}] \end{array}$$

where α_H_ = $K_{2}^{\text{H}}$[H^+^]+$K_{2}^{\text{H}}$$K_{3}^{\text{H}}$[H^+^]^2^+…+$K_{2}^{\text{H}}$$K_{3}^{\text{H}}$$K_{4}^{\text{H}}$$K_{5}^{\text{H}}$$K_{6}^{\text{H}}$[H^+^]^5^. Under the conditions used in our experiments (pH = 4.0–6.0), hydrolysis of Fe^III^ and Ga^III^ may occur by formation of [M(OH)]^2+^, [M(OH)_2_]^+^ and M(OH)_3_ species, i.e., OH^−^ ions may compete with H_x_TRAP^(x−6)^ for formation of [^*^M(HTRAP)]^2−^ intermediate. Considering the hydrolysis of Fe^III^ and Ga^III^, the total metal ion concentration can be expressed by Equation (12).

(12) [MIII]t=[M*(HTRAP)]+[M(OH)]+[M(OH)2]+[M(OH)3]              +[MIII]

By taking into account the stability constant of the [^*^M(HTRAP)]^2−^ intermediate [Equation (9)] and the equilibrium constants characterizing the hydrolysis of Fe^III^ and Ga^III^ (β_x_ = [M(OH)_x_][H^+^]^x^/[M^III^], x = 1, 2, and 3), the total metal ion concentration can be expressed as follows:

(13) [MIII]t=[MIII](1+K*M(HTRAP)[TRAP]free1+αH+β1OH[H+]+β2OH[H+]2+β3OH[H+]3)=[MIII](1+K*M(HTRAP)[TRAP]free1+αH+αOH)

wherein α_OH_ = $\beta_{1}^{\text{OH}}$/[H^+^] +$\beta_{2}^{\text{OH}}$/[H^+^]^2^+ $\beta_{3}^{\text{OH}}$/[H^+^]^3^ (log$\beta_{1}^{\text{OH}}$ = −2.19; log$\beta_{2}^{\text{OH}}$ = −5.67 and log$\beta_{3}^{\text{OH}}$ = −12.0 for Fe^III^ and log$\beta_{1}^{\text{OH}}$ = −2.97; log$\beta_{2}^{\text{OH}}$ = −5.92 and log$\beta_{3}^{\text{OH}}$ = −8.2 for Ga^III^ ion; Baes and Mesmer, 1976). Considering the protonation constants of TRAP^6−^ (Table 1), the stability constant of the [^*^M(HTRAP)]^2−^ intermediate [Equation (9)], the total concentration of the M^III^ ion [Equation (13)], the concentration of the non-complexed TRAP_free_ ligand [Equation (11) and Equation (10)], the pseudo-first order rate constant can be expressed by Equation (14).

(14) kobs=kfK*M(HTRAP)[TRAP]free1+αH1+K*M(HTRAP)[TRAP]free1+αH+αOH

The pseudo-first-order rate constants determined at various pH and [TRAP]_t_ values (Figures 3, 4) were fitted to Equation (14) and the stability constant of the [^*^M(HTRAP)]^2−^ intermediates [^*^K_M(HL)_] and the *k*_f_ rate constants were calculated.

The stability constants of the [^*^Fe(HTRAP)]^2−^ and [^*^Ga(HTRAP)]^2−^ intermediates [log^*^K_M(HL)_] are 9.9 ± 0.1 and 10.4 ± 0.1, respectively. The log^*^K_M(HL)_ values of the [^*^Fe(HTRAP)]^2−^ and [^*^Ga(HTRAP)]^2−^ intermediates are significantly higher than those of the mono-protonated [^*^Ga(HNOTA)]^+^ (log^*^K_Ga(HL)_ = 4.2), (Morfin and Toth, 2011) [^*^Ce(HNOTA)]^+^ (log^*^K_Ce(HL)_ = 3.2), (Brucher and Sherry, 1990) [^*^Gd(HNOTA)]^+^ (log^*^K_Gd(HL)_ = 3.6) (Brucher and Sherry, 1990) and [^*^Er(HNOTA)]^+^ (log^*^K_Er(HL)_ = 3.8) (Brucher and Sherry, 1990) intermediates. In the [^*^Fe(HTRAP)]^2−^ and [^*^Ga(HTRAP)]^2−^ intermediates, Fe^III^ and Ga^III^ are presumably coordinated by three carboxylate and three phosphinate oxygen donor atoms, whereas the metal ions in [^*^M(HNOTA)]^+^ intermediates are coordinated by three carboxylate oxygen donor atoms, resulting in lower log^*^K_M(HL)_ values.

**Figure 5:**
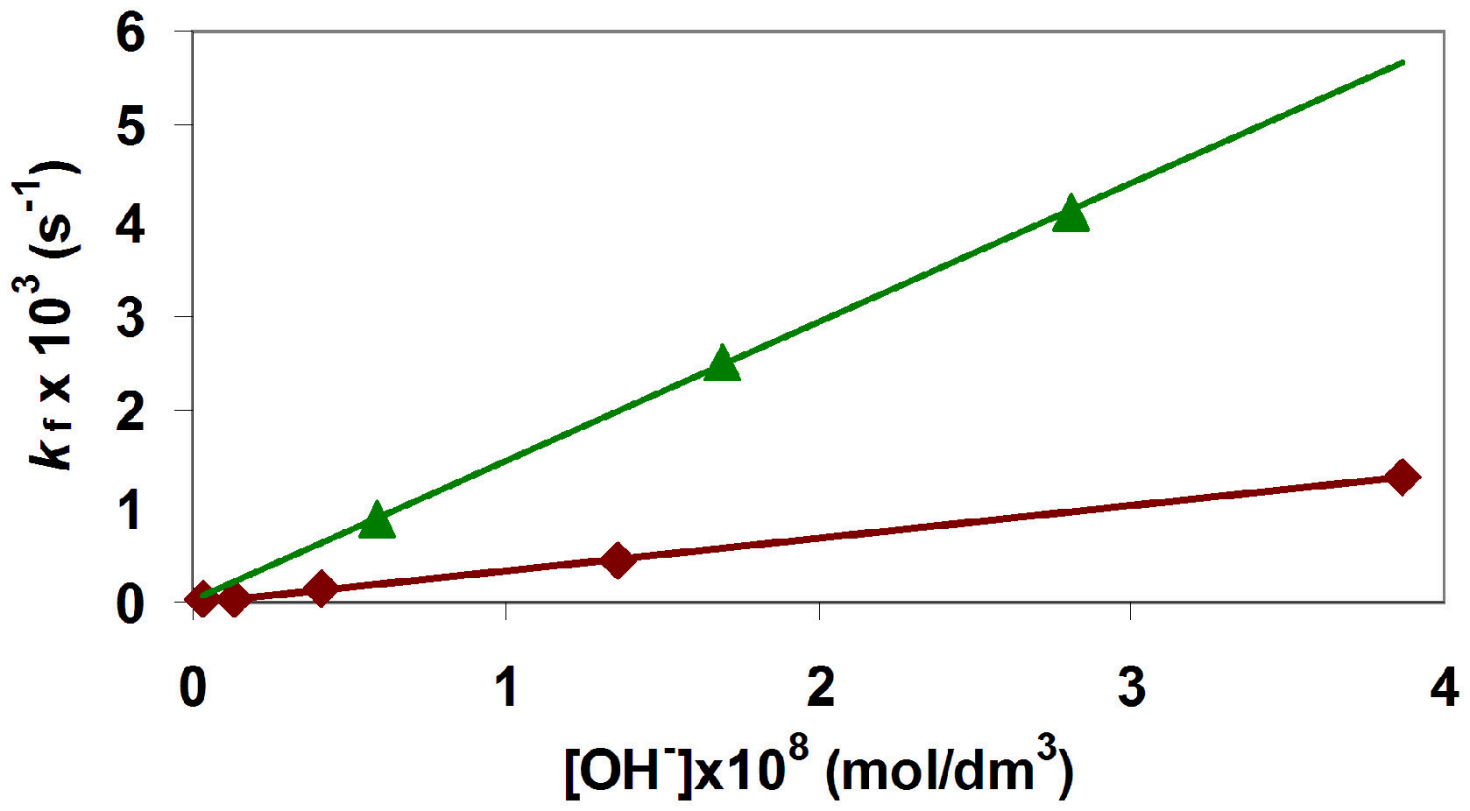
Formation rate constants **(*k***_f_**)** for [$\text{Ga}(\text{TRAP}){]}^{3-}$ and [$\text{Fe}(\text{TRAP}){]}^{3-}$ as a function of [OH^−^].

The calculated *k*_f_ rate constants obtained for formation of [Fe(TRAP)]^3−^ and [Ga(TRAP)]^3−^ complexes are shown in Figure 5 as functions of [OH^−^]. Kinetic data in Figure 5 show that the *k*_f_ values increase monotonously with increasing OH^−^ concentration, while interception of linear extrapolations at the origin indicates that under our experimental conditions, deprotonation and transformation of the [^*^M(HTRAP)]^2−^ intermediate to the final [M(TRAP)]^3−^ complex predominantly occurs by an OH^−^-catalyzed pathway. The *k*_OH_ rate constants calculated from the slopes of the straight lines in Figure 5 are shown in Table 3.

**Table 3:** Rate constants characterizing the formation (*k*_OH_) and dissociation (^M(L)OH^*k*_OH_, ^M(L)^^(OH)2^*k*_OH_) of [Fe(TRAP)]^3−^, [Ga(TRAP)]^3−^, [Ga(NOTA)], and [Ln(NOTA)] complexes (25°C).

	**Formation kinetics**	**Dissociation kinetics**				
	***k*_OH_ /M^−1^s^−1^**	**^M(L)OH^*k*_OH_/s^−1^**	**^M(L)^^(OH)2^*k*_OH_/s^−1^**	**log*K*_M(L)__(OH)2_**	***k*_d_/s^−1^ at pH = 7.4**	***t*_1/2_/h at pH = 7.4**
[Fe(TRAP)]^3−^	(3.37 ± 0.02) × 10^4^	(4 ± 1) × 10^−7^	(5.2 ± 0.4) × 10^−4^	13.4 (1)	1.8 × 10^−9^	1.1 × 10^5^
[Ga(TRAP)]^3−^	(1.47 ± 0.02) × 10^5^	(4.3 ± 0.5) × 10^−7^	(3.8 ± 0.2) × 10^−6^	10.9 (1)	1.4 × 10^−9^	1.4 × 10^5^
[Ga(NOTA)]^a^	1.14 × 10^5^	–	–	–	–	–
[Ce(NOTA)]^b^	6.3 × 10^7^	–	–	–	–	–
[Gd(NOTA)]^b^	7.1 × 10^7^	–	–	–	–	–
[Er(NOTA)]^b^	5.5 × 10^7^	–	–	–	–	–

a Ref. (Morfin and Toth, 2011);

b *Ref. (Brucher and Sherry, 1990)*.

Comparison of the *k*_OH_ rate constants presented in Table 3 shows that the formation rates of [Ga(TRAP)]^3−^ and [Ga(NOTA)] complexes in this pathway are similar and about two orders of magnitude lower than those of [Ln(NOTA)] complexes. The results of the labeling experiments with the TRAP and NOTA chelates of ^68^Ga^III^ at identical conditions (10 nM ligand, pH = 3.3 and 20 °C) shows that the formation rate of [^68^Ga(TRAP)]^3−^ surpasses that of [^68^Ga(NOTA)] (Notni et al., 2010). The faster formation of [^68^Ga(TRAP)]^3−^ can be explained by the higher stability [^*^K_Ga(HL)_] and consequently the higher concentration of the kinetically active [^*^Ga(HTRAP)]^2−^ intermediate that results in the more rapid formation of [^68^Ga(TRAP)]^3−^ in the same labeling condition. On the other hand, the formation rate of [Fe(TRAP)]^3−^ is about 3 times lower than that of Ga(TRAP), which allows to perform selective labeling of TRAP with ^68^Ga^III^ even in presence of Fe^III^ contaminations in the eluate.

### Kinetic inertness and transchelation reaction of complexes

**Figure 6:**
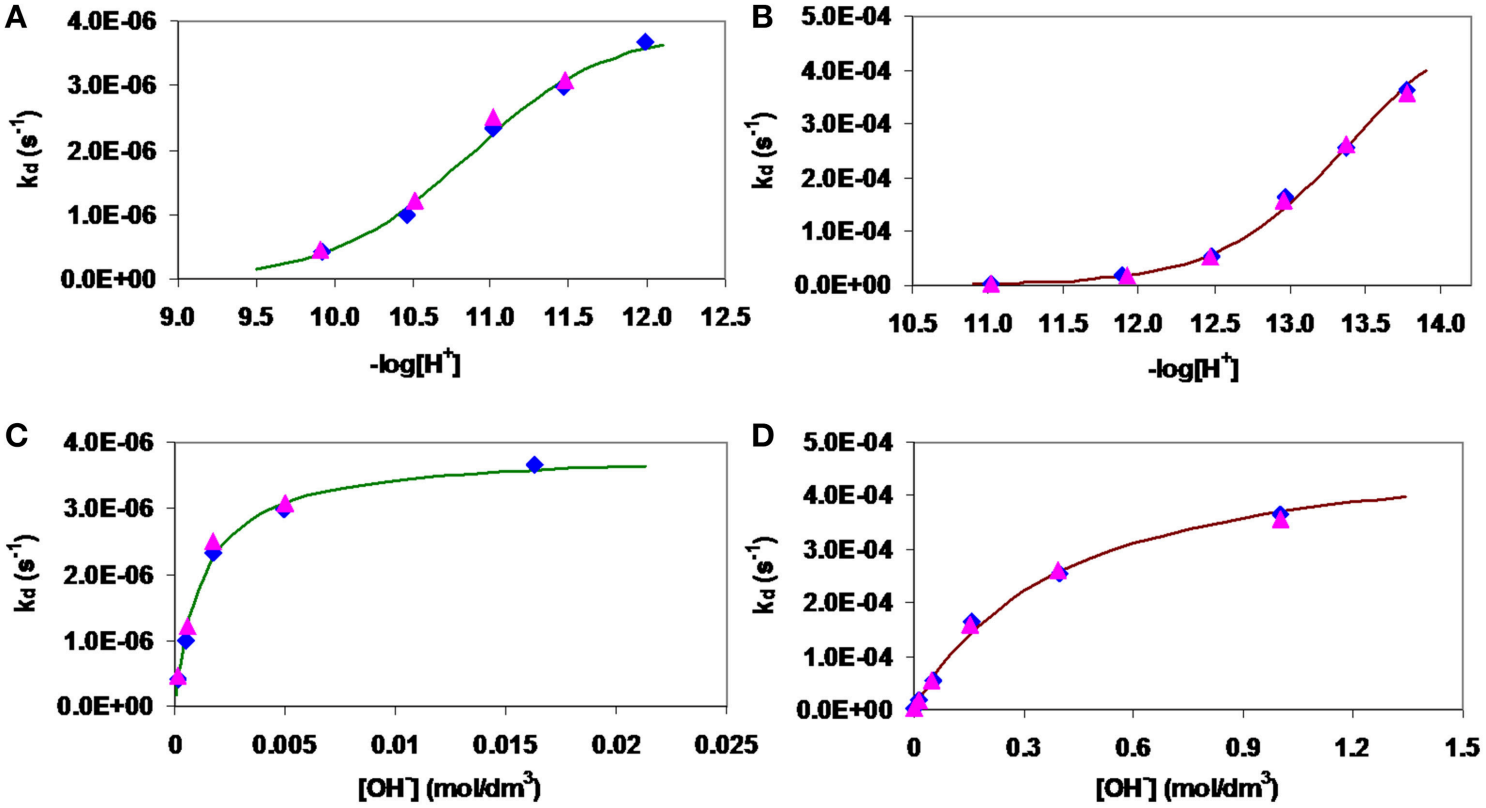
Pseudo-first-order rate constants (*k*_d_) of the ligand exchange reactions of [$Ga{(TRAP)}^{3-}$] (A,C) and [${\text{Fe(TRAP)}}^{3-}$] (B,D) wih H_x_HBED^(x−4)^ as a function of –log[H^+^] and [OH^−^] (x = 0,1, and 2). Solid lines and symbols represent calculated and experimental *k*_d_ pseudo-first-order rate constants, respectively. ([Ga(TRAP)] = [Fe(TRAP)] = 0.2 mM, [H_x_HBED] = 2.0 mM (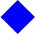), and 4.0 mM (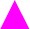), 0.15 M NaCl, 25°C).

In order to compare the kinetic inertness, the rates of transchelation reactions of Fe(TRAP) and Ga(TRAP) complexes with H_x_HBED^x−4^ (x = 0, 1 and 2) ligand were studied because of the high stability of the [Fe(HBED)]^−^ and [Ga(HBED)]^−^ complexes [log*K*_Fe(HBED)_ = 39.01, log*K*_Ga(HBED)_ = 38.51, 0.1 M KCl, 25°C, (Ma et al., 1994)]. The transchelation reactions were followed by spectrophotometry on the absorption band of the forming [Fe(HBED)]^−^ and [Ga(HBED)]^−^ complexes in the pH ranges 11.0–14.0 and 9.0–12.0, respectively. The absorption spectra of the protonated HHBED^3−^ and H_2_HBED^2−^ ligands and [Ga(HBED)]^−^ complex are different, whereas that of the deprotonated HBED^4−^ ligand and [Ga(HBED)]^−^ complex are very similar. Therefore, the transchelation reactions of [Ga(TRAP)]^3−^ with HHBED^3−^ and H_2_HBED^2−^ could be monitored by spectrophotometry only up to pH = 12.0 (HBED^4−^: log$K_{1}^{\text{H}}$ = 12.57(4), log$K_{2}^{\text{H}}$ = 11.41(3), log$K_{3}^{\text{H}}$ = 8.22(5), log$K_{4}^{\text{H}}$ = 4.73(6) and log$K_{5}^{\text{H}}$ = 1.45(6), 0.15 M NaCl, 25°C). Some characteristic absorption spectra of [Fe(TRAP)]^3−^-H_x_HBED^x−4^ and [Ga(TRAP)]^3−^–H_x_HBED^x−4^ (x = 0, 1 and 2) reacting systems are shown in Figures S11, S12, respectively. The transchelation reactions can be described by Equation (15)

(15) $$\begin{array}{l} {\left[\text{M}(\text{TRAP})\right]}^{\text{3}-}+{\text{H}}_{\text{x}}{\text{HBED}}^{\left(\text{x}-\text{4}\right)}⇌{\left[\text{M}(\text{HBED})\right]}^{-}\\ +{\text{H}}_{\text{y}}{\text{TRAP}}^{\left(\text{y}-\text{6}\right)}\text{+}(\text{x}-\text{y}){\text{H}}^{\text{+}} \end{array}$$

wherein M^III^ is Fe^III^ or Ga^III^, x = 0, 1 and 2 and y = 0 and 1. The rates of the transchelation reactions have been studied in the presence of 10- and 20-fold excess of H_x_HBED^(x−4)^, so a pseudo-first order kinetic model can be applied and the rates of reaction Equation (15) can be expressed by Equation (16):

(16) $$-\frac{\text{d}{\left[\text{M}(\text{TRAP})\right]}_{\text{t}}}{\text{dt}}={\text{k}}_{\text{d}}{\left[\text{M}(\text{TRAP})\right]}_{\text{t}}$$

wherein *k*_d_ is a pseudo-first-order rate constant, [M(TRAP)]_t_ is the total concentration of [Fe(TRAP)]^3−^ and [Ga(TRAP)]^3−^ complexes. The pseudo-first-order rate constants (*k*_d_) characterizing the transchelation reactions of [Fe(TRAP)]^3−^ and [Ga(TRAP)]^3−^ with H_x_HBED^(x−4)^ at different –log[H^+^] and [OH^−^] values are shown in Figure 6. The kinetic data presented in Figure 6 show that the *k*_d_ values are independent of the concentration of H_x_HBED^(x−4)^ and increase with –log[H^+^] and [OH^−^], indicating that the rate-determining step of the transchelation reactions is the dissociation of the [Fe(TRAP)]^3−^ and [Ga(TRAP)]^3−^ complexes, followed by fast reaction of free Fe^III^ and Ga^III^ with H_x_HBED^(x−4)^. The *k*_d_ values presented in Figure 6 show the similar behavior of [Fe(TRAP)]^3−^ and [Ga(TRAP)]^3−^ complexes in their transchelation reactions. The *k*_d_ vs. –log[H^+^] and *k*_d_ vs. [OH^−^] curves (Figure 6) obtained for [Ga(TRAP)]^3−^ and [Fe(TRAP)]^3−^ reach saturation of the *k*_d_ values at [OH^−^] > 0.015 M and [OH^−^] > 1.0 M, respectively. Based on the species distribution of the Ga^III^-TRAP^6−^ (Notni et al., 2010) and Fe^III^-TRAP^6−^ (Figure 2) systems, the transchelation reaction of [Ga(TRAP)]^3−^ and [Fe(TRAP)]^3−^ with H_x_HBED^(x−4)^ may occur by the spontaneous dissociation of [M(TRAP)]^3−^ (*k*_0_) and [M(TRAP)OH]^4−^ species (^M(L)OH^*k*_OH_), whereas the pH-independent dissociation rate (*k*_d_) of [M(TRAP)]^3−^ under more basic conditions corresponds to formation [*K*_M(L)__(OH)2_, Equation (17)] and slow dissociation of the bis(hydroxo) [M(TRAP)(OH)_2_]^5−^ intermediate.

(17) $$\begin{array}{l} {\left[\text{M}(\text{TRAP}){\left(\text{OH}\right)}_{2}\right]}^{\text{5}-}+{\text{H}}^{+}⇌{\left[\text{M}(\text{TRAP})\text{OH}\right]}^{\text{4}-}\\ K_{\text{M}(\text{L}){\left(\text{OH}\right)}_{\text{2}}}=\frac{\left[\text{M}(\text{TRAP})\text{OH}\right]}{\left[\text{M}(\text{TRAP}){\left(\text{OH}\right)}_{\text{2}}][{\text{H}}^{+}\right]} \end{array}$$

**Scheme 3:**
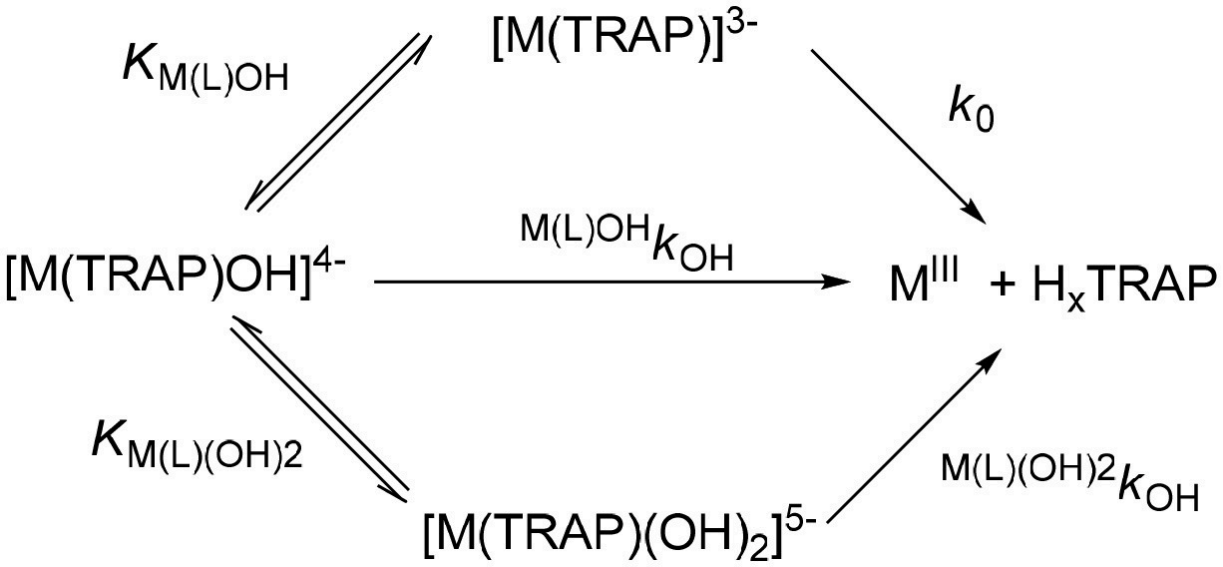
Proposed mechanism of the dissociation of [Fe(TRAP)]^3−^ and [Ga(TRAP)]^3−^ complexes (x = 0 and 1).

It can be assumed that in the [M(TRAP)(OH)_2_]^5−^ intermediate, TRAP^6−^ is coordinating via four donor atoms, whereas the remaining two coordination sites of Ga^III^ and Fe^III^ are occupied by two OH^−^ ions. Hence, a spontaneous dissociation of the [M(TRAP)(OH)_2_]^5−^ intermediates is more probable, which is reflected by the ^M(L)^^(OH)2^*k*_OH_ rate constants. The mechanisms of the transchelation reactions of [Fe(TRAP)]^3−^ and [Ga(TRAP)]^3−^ are summarized in Scheme 3.

By taking into account all possible pathways (Scheme 3), the dissociation rate of [Fe(TRAP)]^3−^ and [Ga(TRAP)]^3−^ can be expressed by Equation (18).

(18) $$\begin{array}{l} -\frac{\text{d}{\left[\text{ML}\right]}_{\text{t}}}{\text{dt}}\text{=}k_{\text{d}}{\left[\text{ML}\right]}_{\text{t}}=k_{\text{0}}[\text{ML}]{+}^{\text{M}(\text{L})\text{OH}}k_{\text{OH}}[\text{M}(\text{L})\text{OH}]\\ {+}^{\text{M}(\text{L}){\left(\text{OH}\right)}_{\text{2}}}k_{\text{OH}}\left[\text{M}(\text{L}){\left(\text{OH}\right)}_{2}\right] \end{array}$$

Considering the total concentrations of [Fe(TRAP)]^3−^ and [Ga(TRAP)]^3−^ ([ML]_t_ = [ML]+[M(L)OH]+[M(L)(OH)_2_]) and the protonation constants of [M(L)OH]^4−^ [*K*_M(L)OH_, Equation (6), Table 2) and [M(L)(OH)_2_]^5−^ intermediates (*K*_M(L)__(OH)2_, Equation (17)], the *k*_d_ pseudo-first-order rate constants presented in Figure 6 can be expressed by Equation (19).

(19) $$k_{d}=\frac{k_{0}K_{\text{M}(\text{L})\text{OH}}[{\text{H}}^{+}]{+}^{\text{M}(\text{L})\text{OH}}k_{\text{OH}}{+}^{M(L){\left(OH\right)}_{2}}k_{OH}{\left(K_{\text{M}(\text{L})\text{OH}}[{\text{H}}^{+}]\right)}^{-1}}{1+{\text{K}}_{\text{M}(\text{L})\text{OH}}[{\text{H}}^{+}]+{\left(K_{\text{M}(\text{L}){\left(\text{OH}\right)}_{\text{2}}}[{\text{H}}^{+}]\right)}^{-1}}$$

wherein *k*_0_, ^M(L)OH^*k*_OH_ and ^M(L)^^(OH)2^*k*_OH_ are the rate constants characterizing the spontaneous dissociation of [M(TRAP)]^3−^, and [M(TRAP)OH]^4−^ complexes and [M(TRAP)(OH)_2_]^5−^ intermediates, whereas *K*_M(L)__(OH)2_ is the equilibrium constant characterizing the formation of the bis(hydroxo) [M(TRAP)(OH)_2_]^5−^ intermediates.

The rate and protonation constants characterizing the transchelation reactions of [Fe(TRAP)]^3−^ and [Ga(TRAP)]^3−^ with H_x_HBED^(x−4)^ have been calculated by fitting the *k*_d_ values presented in Figure 6 to the Equation (19), and the resulting values are shown in Table 3. We obtained a very low value with a large error for *k*_0_; therefore, the spontaneous dissociation of [Fe(TRAP)]^3−^ and [Ga(TRAP)]^3−^ is negligible under our experimental conditions. The ^M(L)OH^*k*_OH_ rate constants characterizing the spontaneous dissociation of [Fe(TRAP)OH]^4−^ and [Ga(TRAP)OH]^4−^ complexes are very similar, which indicates that the kinetic inertness of [Fe(TRAP)OH]^4−^ and [Ga(TRAP)OH]^4−^ are comparable. Interestingly, the *K*_M(L)__(OH)2_ protonation constants indicate that the formation of [Fe(TRAP)(OH)_2_]^5−^ intermediate takes place at significantly higher –log[H^+^] values than that of [Ga(TRAP)(OH)_2_]^5−^. However, the ^M(L)^^(OH)2^*k*_OH_ rate constant of [Fe(TRAP)(OH)_2_]^5−^ intermediate is about two orders of magnitude higher than that of [Ga(TRAP)(OH)_2_]^5−^, which indicates the considerably lower kinetic inertness of the [Fe(TRAP)(OH)_2_]^5−^ intermediate.

In order to compare the kinetic inertness directly, the half-lifes (*t*_1/2_ = ln2/*k*_d_) of the dissociation reactions of [Fe(TRAP)]^3−^ and [Ga(TRAP)]^3−^ at pH = 7.4 have been calculated, utilizing the rate and equilibrium constants presented in Table 3. The *t*_1/2_ values of Fe(TRAP) and Ga(TRAP) are 1.1 × 10^5^, and 1.4 × 10^5^ h, respectively, which indicates a similar kinetic inertness of [Fe(TRAP)]^3−^ and [Ga(TRAP)]^3−^ due to comparable ^M(L)OH^*k*_OH_ rate constants of the [Fe(TRAP)OH]^4−^ and [Ga(TRAP)OH]^4−^ complexes. On the other hand, reliability of our kinetic data is supported by a good agreement of the dissociation half-life for [Ga(TRAP)]^3−^ at pH = 11 determined in this study (*t*_1/2_ = 86 h) with the literature value of *t*_1/2_ ≈ 60 h (Notni et al., 2010).

## Conclusion

Due to the availability of ^68^Ge/^68^Ga generators, recent years have seen an ever-growing interest in the radionuclide ^68^Ga^III^ for PET examinations. The corresponding radiopharmaceuticals generally contain ^68^Ga^III^ in form of chelates, for which purpose dedicated bifunctional chelators are usually conjugated to biological targeting vectors. The carrier-free ^68^Ga^III^ obtained by acidic elution from the generator may contain some metal ions as impurities in trace amounts. These metal ions, like Ti^IV^, Fe^III^, Cu^II^, and Zn^II^, may compete with the ^68^Ga^III^ for the chelator's binding sites. Hence, knowledge of the possible interactions of these ions and Ga^III^ with chelates are highly important.

In this work, the interaction of Ga^IIII^ and Fe^III^ ions with H_6_TRAP, a phosphinic acid analog of H_3_NOTA, were studied and compared. The stability constants of the [Ga(TRAP)]^3−^ and [Fe(TRAP)]^3−^ complexes were found to be very similar, as are their very low dissociation rates at physiological pH. The dissociation predominantly occurs via spontaneous dissociation of mono-hydroxo [M(TRAP)OH]^4−^ complexes and bis(hydroxo) [M(TRAP)(OH)_2_]^5−^ intermediates. Similarly to the respective NOTA complexes, formation of Ga(TRAP) and Fe(TRAP) is slow and occurs by formation of the monoprotonated [^*^M(HTRAP)]^2−^ intermediates. The stability of these intermediates is very high, presumably because both the phosphinate and carboxylate groups of the ligand are coordinated. However, although we observed an extraordinary similarity of the thermodynamic and kinetic properties of the Ga(TRAP) and Fe(TRAP) complexes, there is a small but important difference between the two systems: the formation rate of Ga(TRAP) is approximately three times higher than that of the Fe(TRAP), which has implications for the influence of Fe^III^ contaminations on ^68^Ga labeling of TRAP.

Apparently, the previously observed selectivity of TRAP for ^68^Ga^III^ over Fe^III^ is rooted in a totally different mechanism than the preference of TRAP for Ga^III^ over Cu^II^ and Zn^II^ (Simecek et al., 2013). Because Fe(TRAP) is formed more slowly than Ga(TRAP), formation of ^68^Ga(TRAP) is preferred and even a 3-fold excess of Fe^III^ over TRAP does not substantially reduce the labeling yield. However, Fe(TRAP) is kinetically inert, and a higher excess of Fe^III^ ultimately inhibits the ^68^Ga^III^ incorporation due to an irreversible consumption of all available TRAP. On the other hand, the TRAP complexes of Zn^II^ and Cu^II^ are formed much faster but they are not inert (Baranyai et al., 2015). Unlike Fe^III^, TRAP-bound Cu^II^ and particularly Zn^II^ may therefore be readily displaced by Ga^III^ (Simecek et al., 2013), driven by a much higher thermodynamic stability of [Ga(TRAP)]^3−^ as compared to [Zn(TRAP)]^4−^ and [Cu(TRAP)]^4−^ (log*K*_ML_ of 26.24, 16.07, and 19.09, respectively) (Notni et al., 2010; Baranyai et al., 2015). Hence, in contrast to Fe^III^, even high concentrations of Cu^II^ and particularly that of Zn^II^ do not completely inhibit ^68^Ga labeling of TRAP, likewise resulting in a pronounced tolerance of these potential contaminants. We conclude that even a phenomenon of elementary character, namely, the selectivity of TRAP for Ga^III^ which manifests itself in a tolerance of remarkably high concentrations of different metal ion impurities during ^68^Ga^III^ labeling, may rely on a variety of driving forces and molecular properties, thus requiring a detailed investigation of mechanistic details for thorough understanding.

## Author contributions

AV and AF contributed to the equilibrium and kinetic characterizations; AW performed the ligand synthesis; EB, IT, AM, H-JW, JN, and ZB contributed to the evaluation of the physico-chemical parameters and to the manuscript preparation.

### Conflict of interest statement

The authors declare that the research was conducted in the absence of any commercial or financial relationships that could be construed as a potential conflict of interest. The reviewer, FG, and handling Editor declared their shared affiliation.
