# Supplementary material for: Equilibrium Thermodynamics, Formation, and Dissociation Kinetics of Trivalent Iron and Gallium Complexes of Triazacyclononane-Triphosphinate (TRAP) Chelators: Unraveling the Foundations of Highly Selective Ga-68 Labeling
Source: Front Chem. 2018 May 23;6:170. doi: 10.3389/fchem.2018.00170 (PMC5974124; doi:10.3389/fchem.2018.00170)
Supplement: Supplementary file 1 [file Data_Sheet_1.doc]

Supplementary Material

# Equilibrium thermodynamics, formation and dissociation kinetics of trivalent iron and gallium complexes of triazacyclononane-triphosphinate (TRAP) chelators: Unraveling the foundations of highly selective 68Ga labeling

Adrienn Vágner,^1^, *^#^* Attila Forgács,^1^*^,#^* Ernő Brücher,^1^ Imre Tóth,^1^ Alessandro Maiocchi,^2^ Alexander Wurzer,^3^ Hans-Jürgen Wester,^3^ Johannes Notni^3^ and Zsolt Baranyai^1,2*^

*^1^Department of Inorganic and Analytical Chemistry, University of Debrecen, Debrecen, Egyetem tér 1, H-4032, Hungary*

*^2^Bracco Imaging spa, Bracco Research Centre, Via Ribes 5, 10010 Colleretto Giacosa (TO), Italy*

*^3^Radiopharmaceutical Chemistry, Technische Universität München, Walther-Meissner-Str. 3, 85748 Garching, Germany*

*# These authors contributed equally.*

*** Correspondence:** Zsolt Baranyai,

Bracco Imaging – CRB/Trieste,

Area Science Park. Ed. Q, SS 14, km 163.5, I-34149,

Basovizza Trieste, Italy,

tel. +39-040-3757842,

fax +39-040-3757831,

E-mail: [zsolt.baranyai@bracco.com](mailto:zsolt.baranyai@bracco.com)

# Stability and protonation constants characterizing Fe^III^-Bha system

Hydroxamic acids form very stable complexes with Fe^III^ ion. Consequently, the determination of the equilibrium constants characterizing the species formed in the Fe^III^ – HBha system are difficult by pH-potentiometric studies only. Fortunately, the interaction between Fe^III^ ion and HBha can be monitored on the charge transfer absorption band in the wavelength range 400 – 800 nm. In the first set of spectrophotometric experiments the absorption spectra of individual samples of Fe^III^-HBha system were recorded in the [H^+^] range 0.04 - 1.0 mM ([Fe^III^] = 0.2 mM, [HBha] = 2.0 mM). The absorption spectra of Fe^III^-Bha systems obtained in the [H^+^] range 0.04 - 1.0 mM are shown in Figure S1. In the second set of the spectrophotometric measurements, the absorption spectra of Fe^III^-HBha systems were recorded in the pH range 1.7 – 11.0 ([Fe^III^] = 0.1 – 0.3 mM, [HBha] = 2.0 mM). The absorption spectra Fe^III^-HBha systems recorded in the pH range 1.7 - 11.0 are presented in Figures S2 – S4. Figure S1 shows that the absorbance values increase with the decrease of [H^+^] in the [H^+^] range 0.20 – 1.0 M. The increase of the absorbance values can be interpreted by the formation of [Fe(Bha)]^2+^ complex characterized by λ_max_= 510 nm wavelength. In the [H^+^] range 0.04 – 0.20 M, a minimal shift of λ_max_ to the lower wavelengths indicates the appearance of [Fe(Bha)_2_]^+^ in low concentration under these conditions. Figures S2 – S4 show significant shifts in λ_max_ and increase in absorbance values with the appearance of two well defined isosbestic points at λ_max_= 560 nm at lower pH and at λ_max_= 490 nm at higher pH in the pH range 1.7 – 6.5. The presence of two isosbestic points can be interpreted by the formation of [Fe(Bha)_2_]^+^ and [Fe(Bha)_3_] complexes. In the pH range 6.5 – 11.0, the absorbance values of Fe^III^-HBha systems decrease with the increase of pH, which can be interpreted by the formation of [Fe(Bha)_2_(OH)_2_]^-^ species via the substitution of one of the Bha^-^ ligand with two OH^-^ ion in [Fe(Bha)_3_] complex.


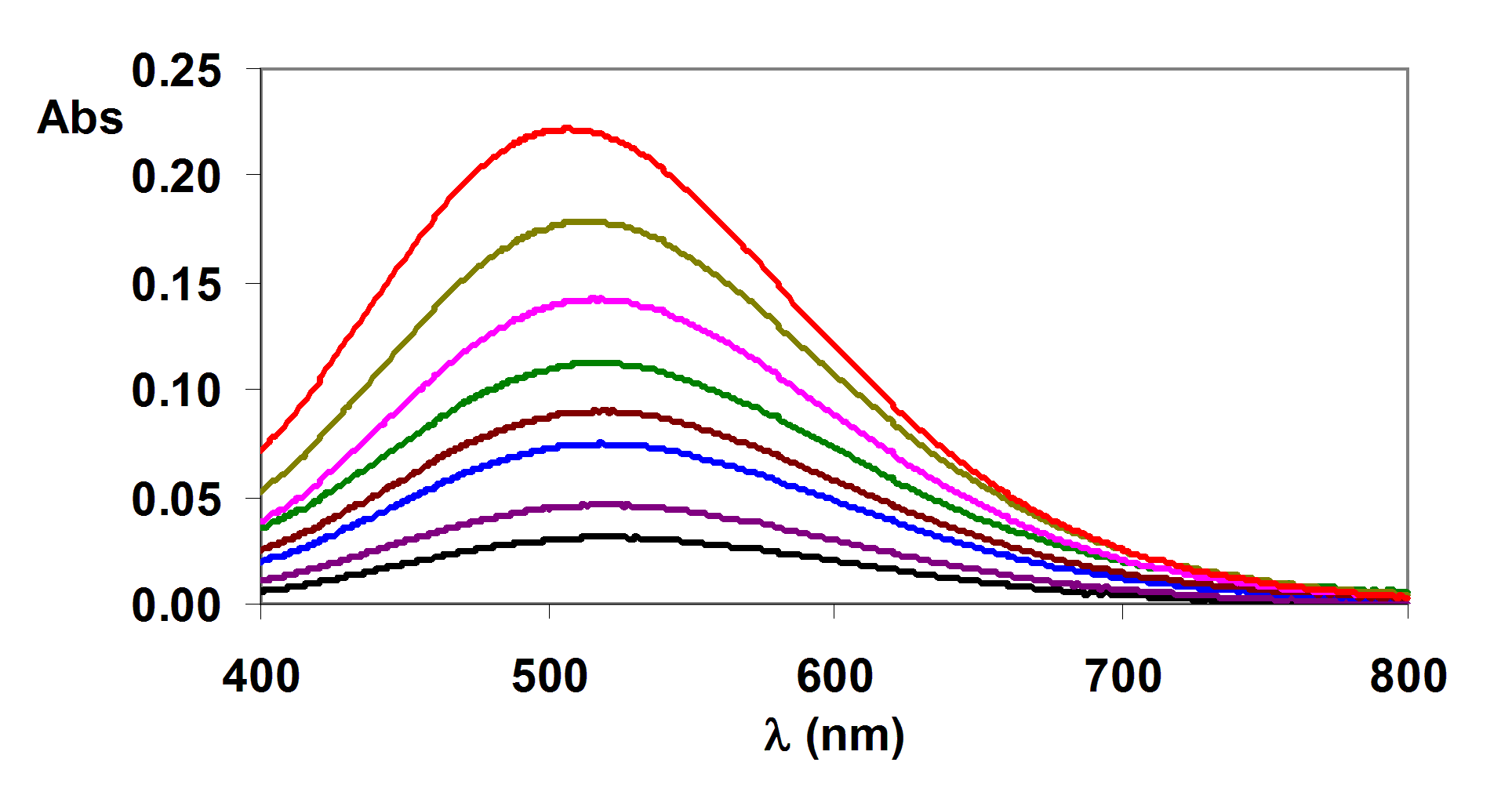


**Figure S1.** Absorption spectra of the Fe^III^ - HBha systems. ([Fe^III^] = 0.2 mM, [HBha] = 2.0 mM, [H^+^] = **1.00 M**, **0.75 M**, **0.50 M**, **0.40 M**, **0.32 M**, **0.20 M**, **0.10 M** and **0.04 M**, [H^+^]+[Na^+^] = 0.15 M in the last two samples, 25 °C)


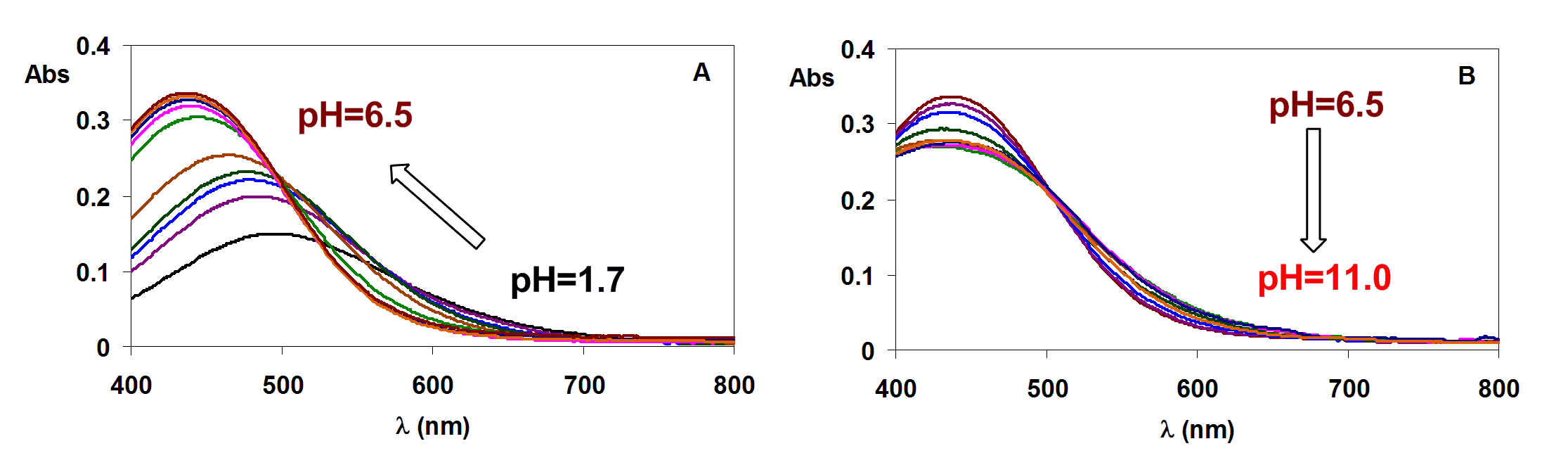


**Figure S2.** Absorption spectra of the Fe^III^ - HBha systems in the pH ranges 1.7 – 6.5 (**A**) and 6.5 – 11.0 (**B**). ([Fe^III^] = 0.1 mM, [HBha] = 2.0 mM, 0.15 M NaNO_3_, 25 °C)


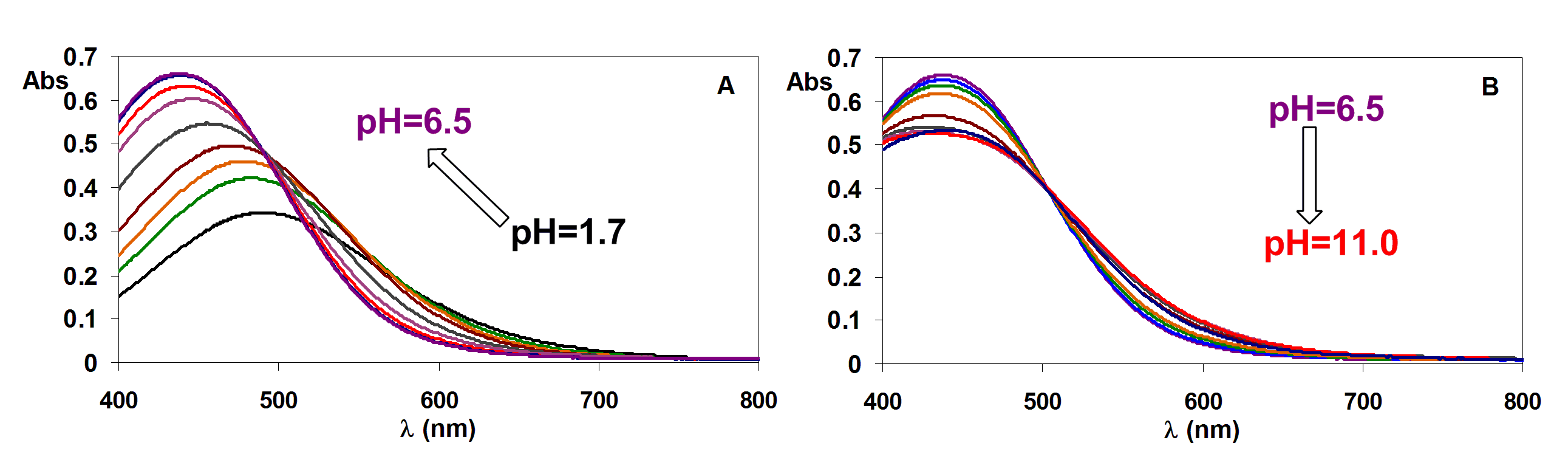


**Figure S3.** Absorption spectra of the Fe^III^ - HBha systems in the pH ranges 1.7 – 6.5 (**A**) and 6.5 – 11.0 (**B**). ([Fe^III^] = 0.2 mM, [HBha] = 2.0 mM, 0.15 M NaNO_3_, 25 °C)


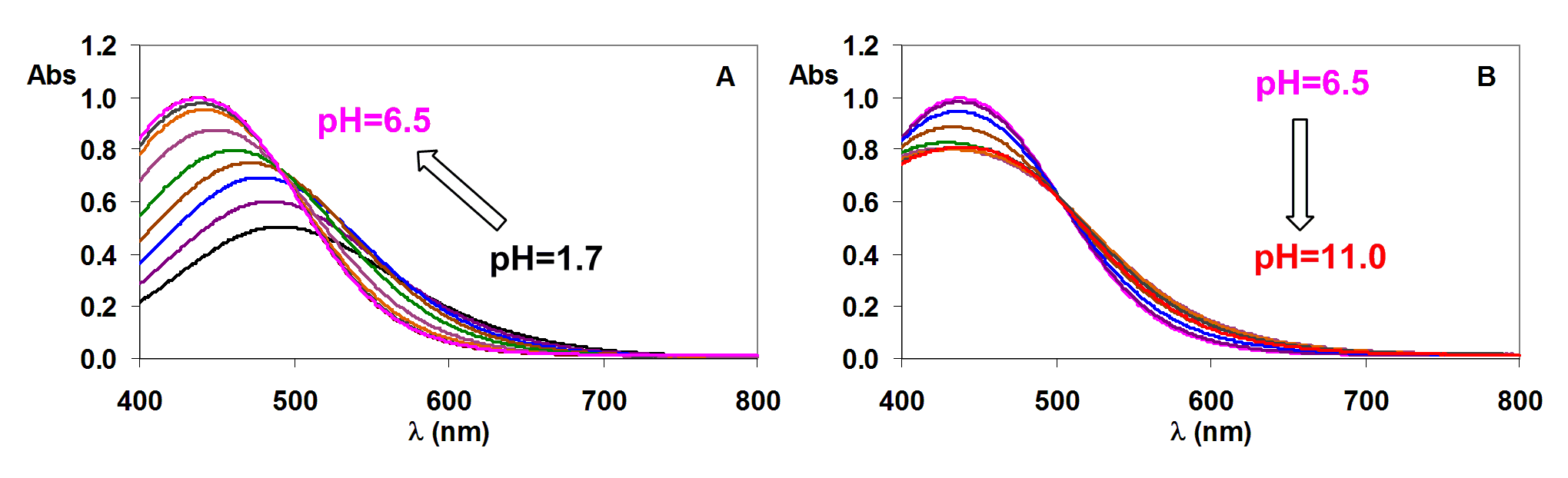


**Figure S4.** Absorption spectra of the Fe^III^ - HBha systems in the pH ranges 1.7 – 6.5 (**A**) and 6.5 – 11.0 (**B**). ([Fe^III^] = 0.3 mM, [HBha] = 2.0 mM, 0.15 M NaNO_3_, 25 °C)

Using all the data obtained by the spectrophotometric and pH-potentiometric measurements the equilibrium constants characterizing the formation of [Fe(Bha)]^2+^ (Eq. (1)), [Fe(Bha)_2_]^+^ (Eq. (2)), [Fe(Bha)_3_] (Eq. (3)) and [Fe(Bha)_2_(OH)_2_]^-^ (Eq. (4)) species were calculated by taking into account the known hydrolysis constants of the free Fe^3+^ ion ([Fe(OH)]^2+^:log*β*_1_^OH^ = –2.19; [Fe(OH)_2_]^+^: log*β*_2_^OH^ = –5.67, Fe(OH)_3_: log*β*_3_^OH^ = –12.0 and [Fe(OH)_4_]^-^: log*β*_4_^OH^ = –21.6). Results are summarized in Table 2. Molar absorptivity values of [Fe(Bha)]^2+^, [Fe(Bha)_2_]^+^, [Fe(Bha)_3_] and [Fe(Bha)_2_(OH)_2_]^-^ species and the species distribution of Fe^III^-HBha system are shown in Figures S5 and S6.

Fe^III^ + Bha^-^ [Fe(Bha)]^2+^ *(1)*

$$K_{Fe(Bha)}=\frac{[Fe(Bha)]}{[Fe^{III}][Bha]}$$

[Fe(Bha)]^2+^ + Bha^-^ [Fe(Bha)_2_]^+^ *(2)*

$$K_{Fe(Bha)_{2}}=\frac{[Fe(Bha)_{2}]}{[Fe(Bha)][Bha]}$$

[Fe(Bha)_2_]^+^ + Bha^-^ [Fe(Bha)_3_] *(3)*

$$K_{Fe(Bha)_{3}}=\frac{[Fe(Bha)_{3}]}{[Fe(Bha){}_{2}][Bha]}$$

Fe^III^ + 2Bha^-^ + 2OH^-^ [Fe(Bha)_2_(OH)_2_]^-^ *(4)*

$$\beta_{Fe(Bha)_{2}(OH)_{2}}=\frac{[Fe(Bha)_{2}H_{-2}]}{[Fe^{III}][Bha]^{2}[OH^{-}]^{2}}$$

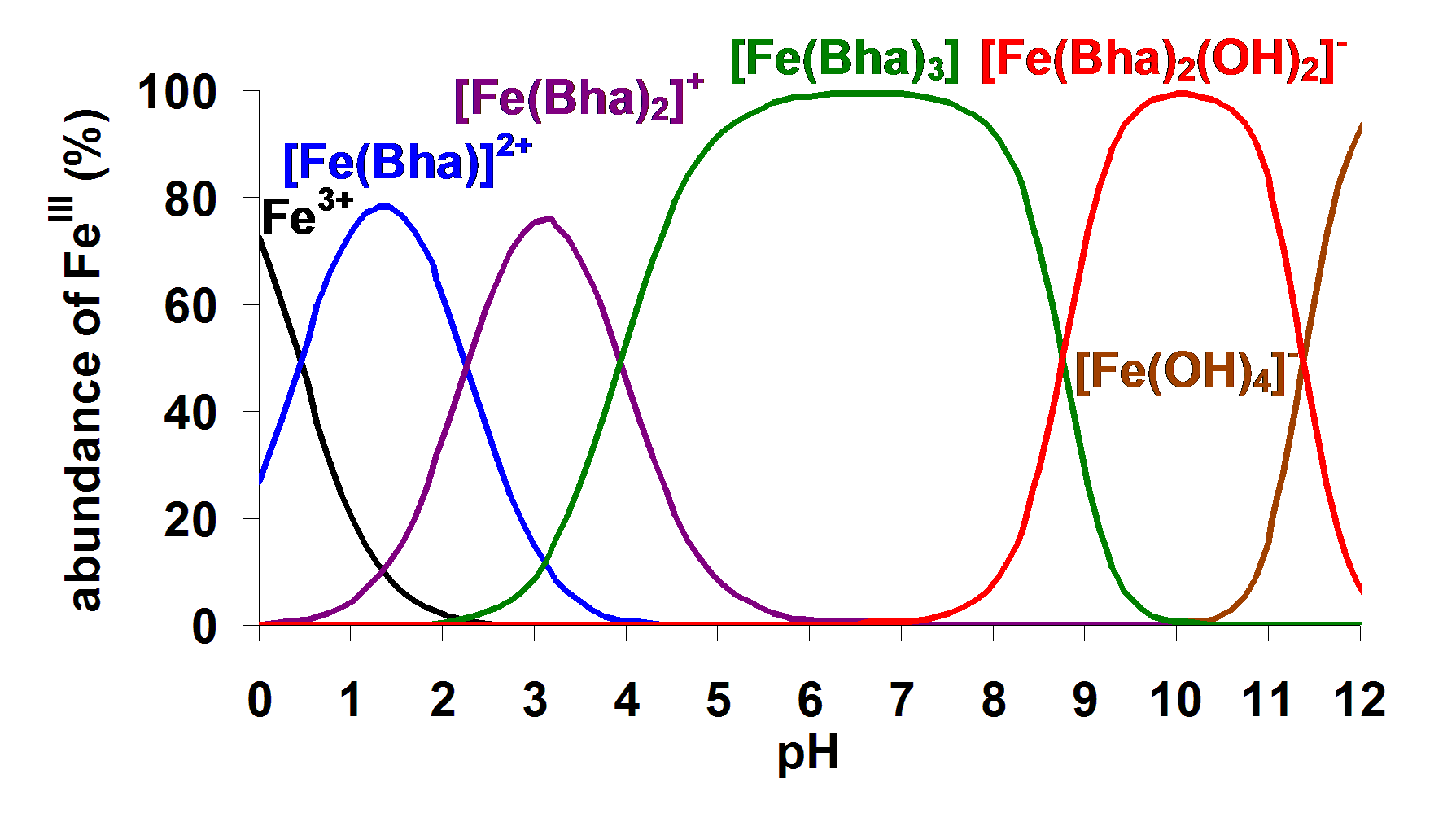


**Figure S5.** Species distribution in the Fe^III^ - HBha system ([Fe^III^] = 0.2 mM, [HBha] = 2.0 mM, 0.15 M NaNO_3_, 25 °C).


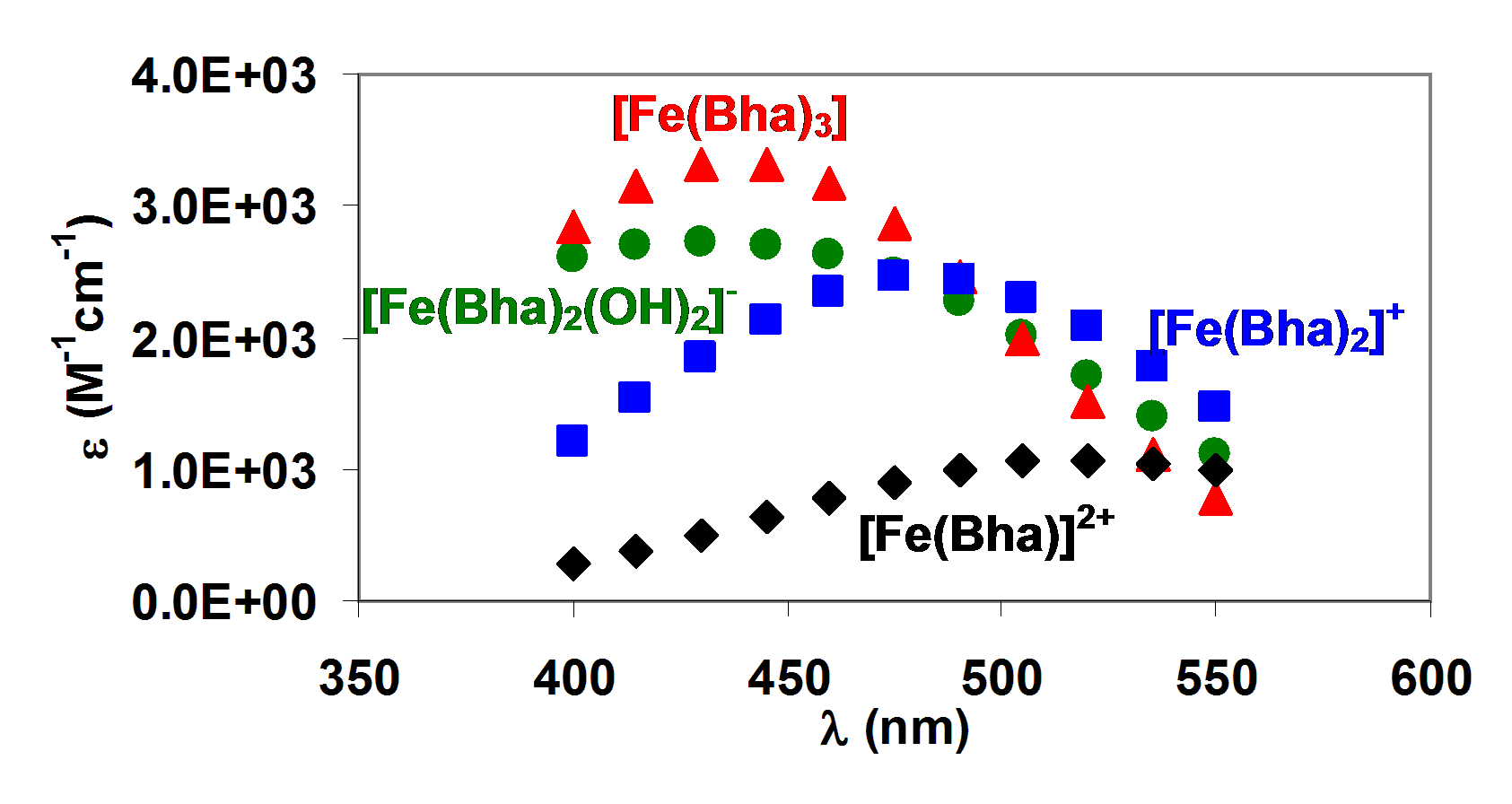


**Figure S6.** Molar absorptivities of [Fe(Bha)]^2+^ (◆), [Fe(Bha)_2_]^+^ (◆), [Fe(Bha)_3_] (▲) and [Fe(Bha)_2_(OH)_2_]^-^ (●) complexes (0.15 M NaNO_3_, 25 °C).

# Equilibrium studies of Fe^III^ – TRAP and Fe^III^-NOTA systems


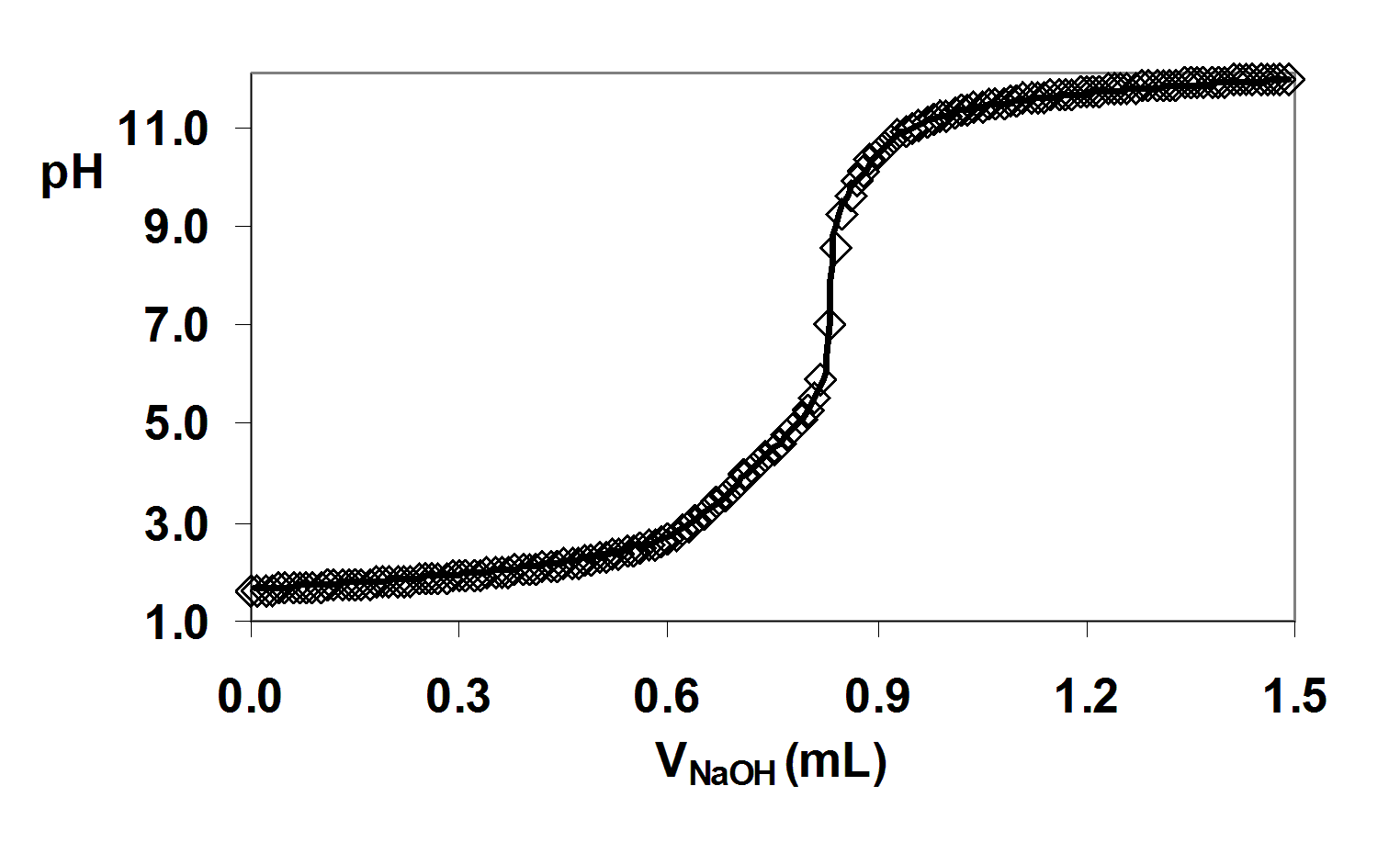


**Figure S7.** pH-potentiometric titration data of [Fe(TRAP)]^3-^ complex. Solid lines and open symbols represent calculated and experimental V_NaOH_ - pH_read_ data pairs, respectively. ([Fe(TRAP)] = 2.1 mM, [HNO_3_] = 29.0 mM, [NaOH] = 0.2090 M, 0.15 M NaNO_3_, 25 °C)


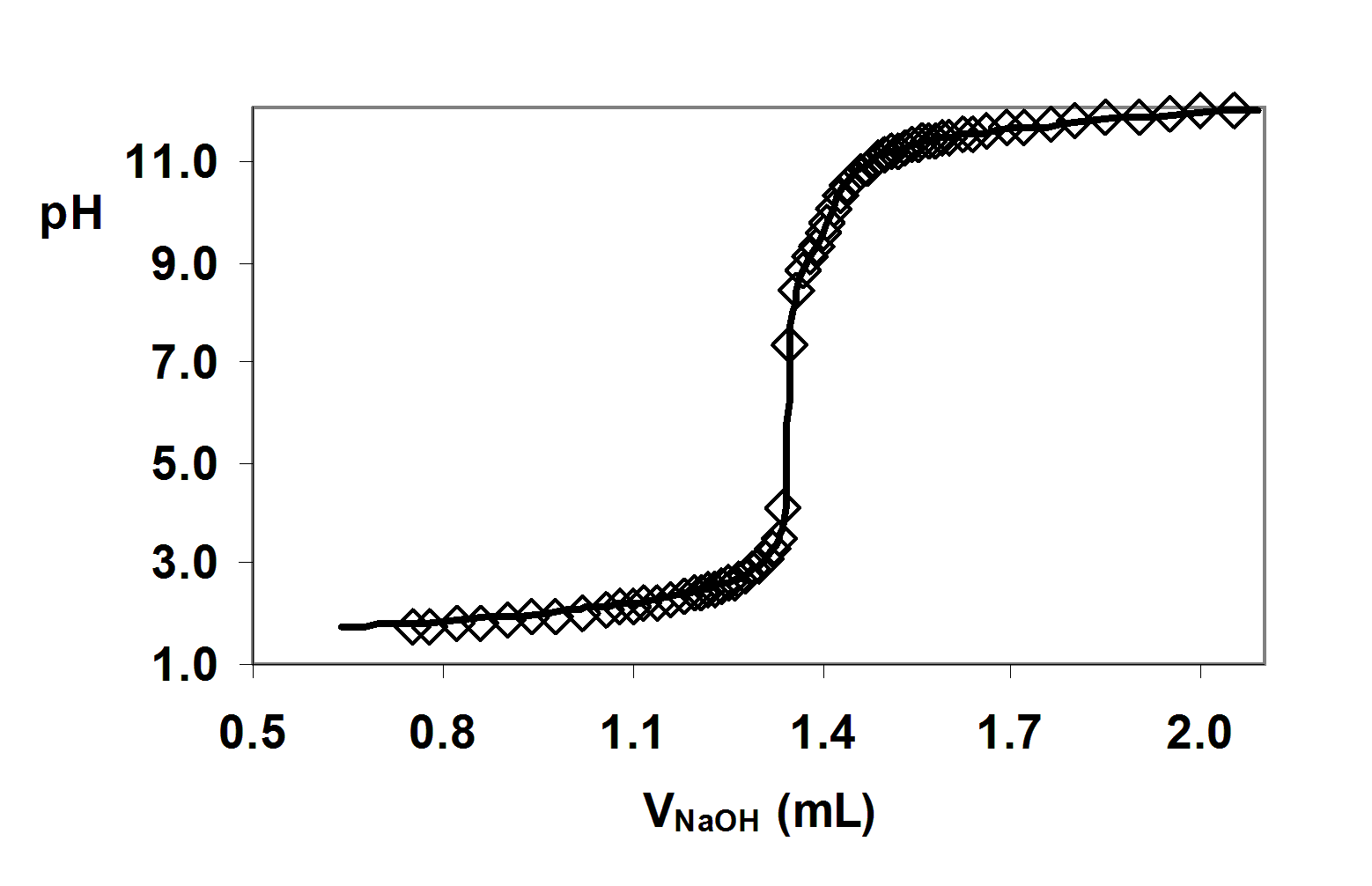


**Figure S8.** pH-potentiometric titration data of [Fe(NOTA)] complex. Solid lines and open symbols represent calculated and experimental V_NaOH_ - pH_read_ data pairs, respectively. ([Fe(NOTA)] = 2.1 mM, [HNO_3_] = 38.9 mM, [NaOH] = 0.1734 M, 0.15 M NaNO_3_, 25 °C)

# Formation kinetics of [Fe(TRAP)]^3-^ and [Ga(TRAP)]^3-^ complexes


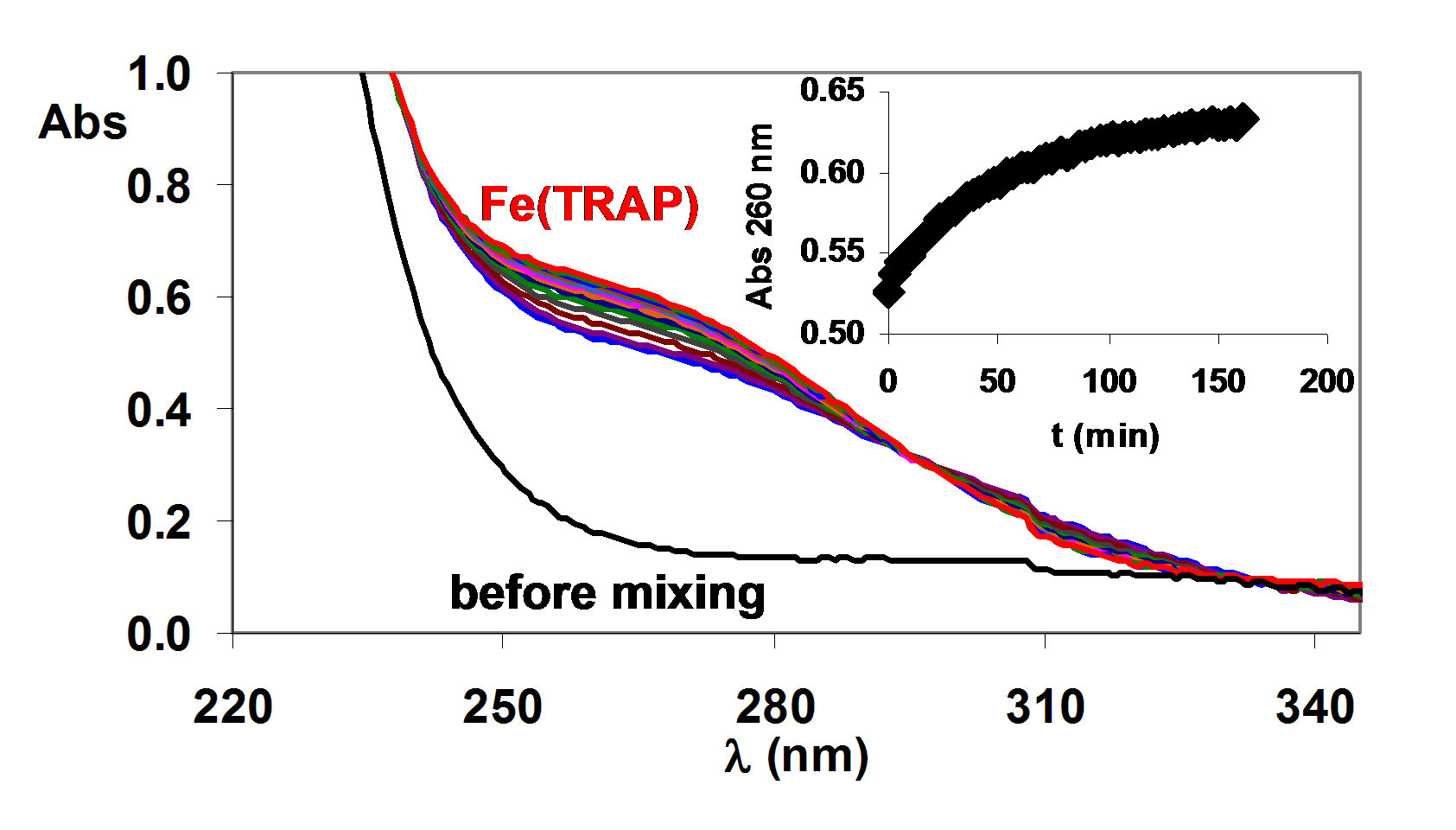


**Figure S9.** Absorption spectra of the Fe^III^-HTRAP^5-^ reacting system before and after the mixing of reactants ([Fe^III^] = 0.1 mM, [H_6_TRAP] = 4.0 mM, pH = 6.0, 0.15 M NaNO_3_, 25°C)


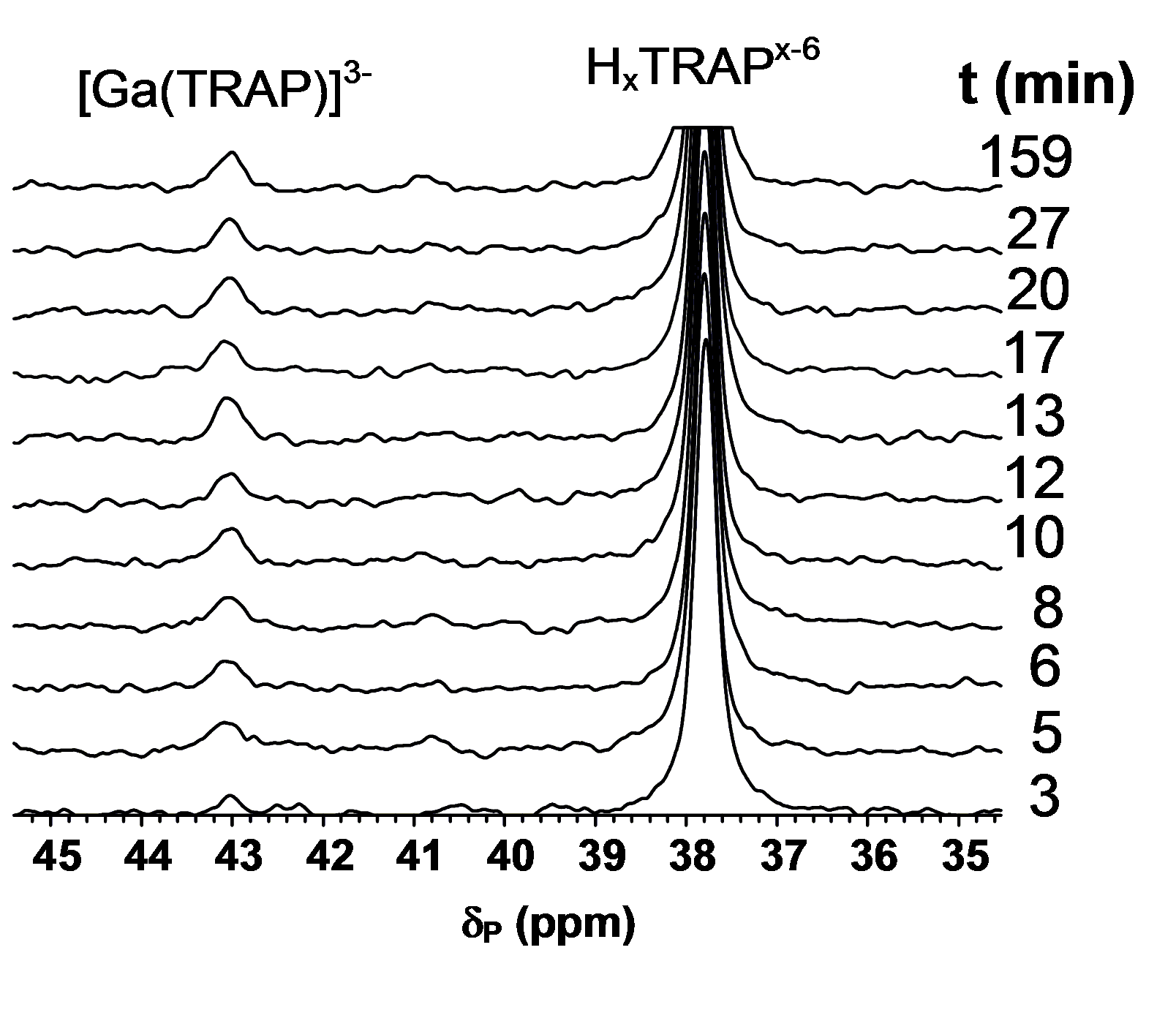


**Figure S10.** ^31^P-NMR spectra of the Ga^III^-HTRAP^5-^ reacting system ([Ga^III^] = 1 mM, [H_6_TRAP] = 10. mM, pH = 6.0, 0.15 M NaNO_3_, 25 °C)

# Kinetic inertness & transchelation


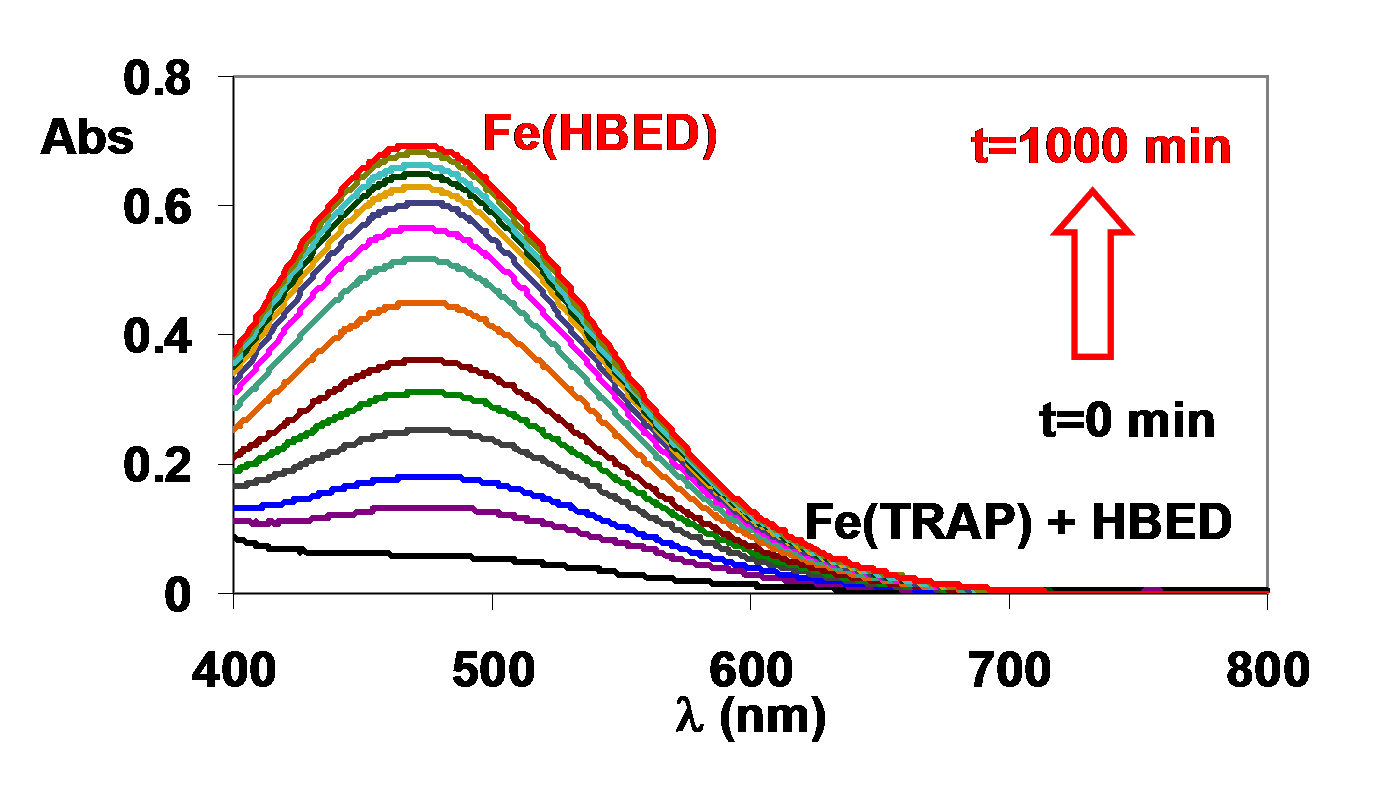


**Figure S11.** Absorption spectra of the [Fe(TRAP)]^3-^ and H_4_HBED reacting system ([Fe(TRAP)] = 0.2 mM, [H_4_HBED] = 2.0 mM, pH=12.7, 0.15 M NaCl, 25 °C)


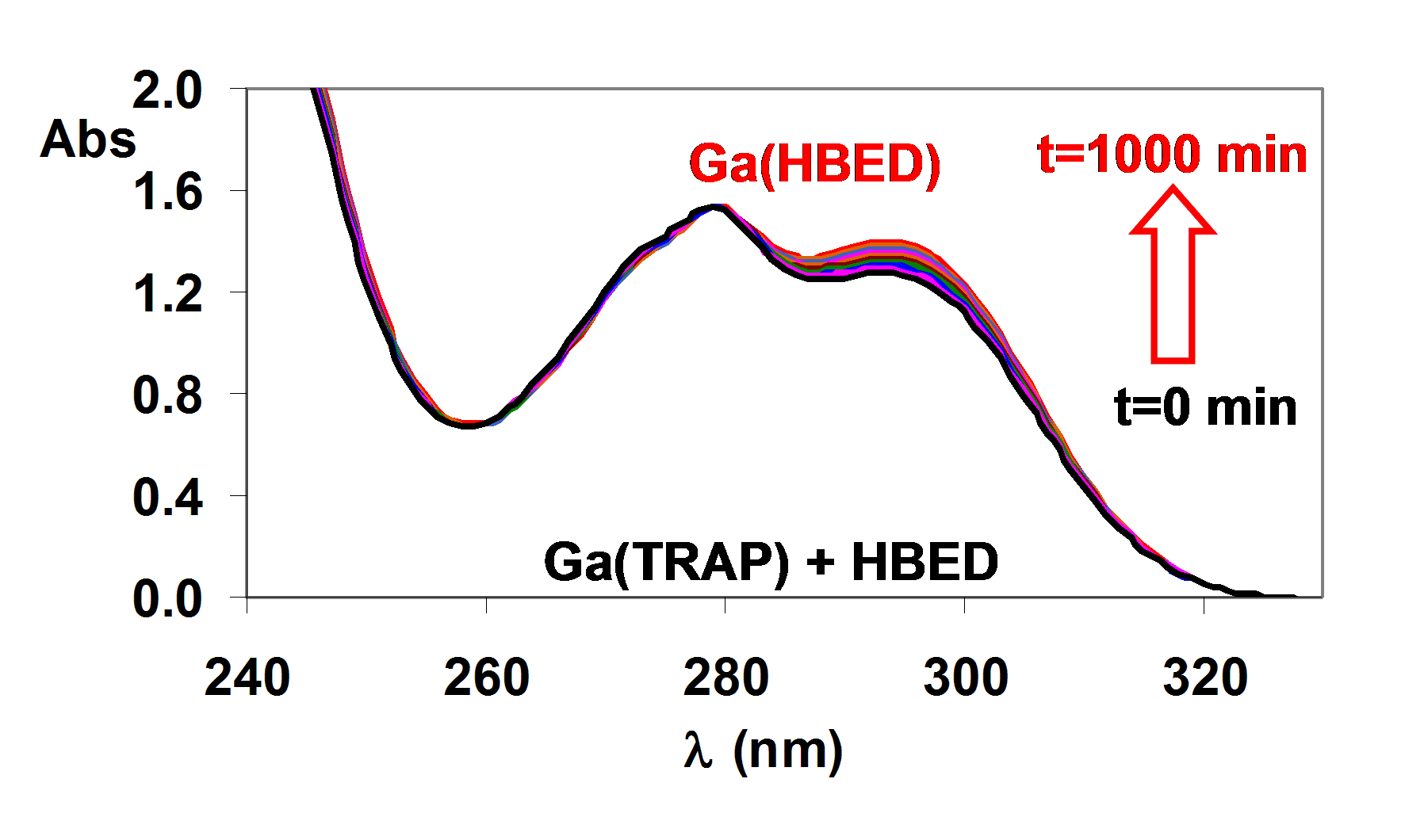


**Figure S12.** Absorption spectra of [Ga(TRAP)]^3-^ – H_4_HBED reacting system ([Ga(TRAP)] = 0.2 mM, [H_4_HBED] = 2.0 mM, pH=11.5, 0.15 M NaCl, 25 °C)

# References

Baes, C.F., and Mesmer, R.E. (1976). *The Hydrolysis of Cations.* New York, London, Sydney, Toronto: John Wiley & Son.

Farkas, E., Kozma, E., Petho, M., Herlihy, K.M., and Micera, G. (1998). Equilibrium studies on copper(II)- and iron(III)-monohydroxamates. *Polyhedron* 17(19)**,** 3331-3342. doi: https://doi.org/10.1016/S0277-5387(98)00113-2.
